# Comparison of Clinical Outcomes of Robot-Assisted, Video-Assisted, and Open Esophagectomy for Esophageal Cancer: A Systematic Review and Meta-analysis

**DOI:** 10.1001/jamanetworkopen.2021.29228

**Published:** 2021-11-01

**Authors:** Michael A. Mederos, Michael J. de Virgilio, Rivfka Shenoy, Linda Ye, Paul A. Toste, Selene S. Mak, Marika S. Booth, Meron M. Begashaw, Mark Wilson, William Gunnar, Paul G. Shekelle, Melinda Maggard-Gibbons, Mark D. Girgis

**Affiliations:** 1 Department of Surgery, David Geffen School of Medicine at UCLA, Los Angeles, California; 2 Veterans Health Administration, Greater Los Angeles Healthcare System, Los Angeles, California; 3 National Clinician Scholars Program, University of California, Los Angeles; 4 Olive View–UCLA Medical Center, Sylmar, California; 5 RAND Corporation, Santa Monica, California; 6 US Department of Veterans Affairs, Washington, DC; 7 Department of Surgery, VA Pittsburgh Healthcare System, Pittsburgh, Pennsylvania; 8 VHA National Center for Patient Safety, Ann Arbor, Michigan; 9 University of Michigan, Ann Arbor

## Abstract

**Importance:**

The utilization of robot-assisted minimally invasive esophagectomy (RAMIE) for esophageal cancer is increasing, despite limited data comparing RAMIE with other surgical approaches.

**Objective:**

To evaluate the literature for clinical outcomes of RAMIE compared with video-assisted minimally invasive esophagectomy (VAMIE) and open esophagectomy (OE).

**Data Sources:**

A systematic search of PubMed, Cochrane, Ovid Medline, and Embase databases from January 1, 2013, to May 6, 2020, was performed.

**Study Selection:**

Studies that compared RAMIE with VAMIE and/or OE for cancer were included.

**Data Extraction and Synthesis:**

Following the Preferred Reporting Items for Systematic Reviews and Meta-Analyses (PRISMA) reporting guideline, data were extracted by independent reviewers. A random-effects meta-analysis of 9 propensity-matched studies was performed for the RAMIE vs VAMIE comparison only. A narrative synthesis of RAMIE vs VAMIE and OE was performed.

**Main Outcomes and Measures:**

The outcomes of interest were intraoperative outcomes (ie, estimated blood loss [EBL], operative time, lymph node [LN] harvest), short-term outcomes (anastomotic leak, recurrent laryngeal nerve [RLN] palsy, pulmonary and total complications, and 90-day mortality), and long-term oncologic outcomes.

**Results:**

Overall, 21 studies (2 randomized clinical trials, 11 propensity-matched studies, and 8 unmatched studies) with 9355 patients were included. A meta-analysis was performed with 9 propensity-matched studies comparing RAMIE with VAMIE. The random-effects pooled estimate found an adjusted risk difference (RD) of −0.06 (95% CI, −0.11 to −0.01) favoring fewer pulmonary complications with RAMIE. There was no evidence of differences between RAMIE and VAMIE in LN harvest (mean difference [MD], −1.1 LN; 95% CI, −2.45 to 0.25 LNs), anastomotic leak (RD, 0.0; 95% CI, −0.03 to 0.03), EBL (MD, −6.25 mL; 95% CI, −18.26 to 5.77 mL), RLN palsy (RD, 0.01; 95% CI, −0.08 to 0.10), total complications (RD, 0.05; 95% CI, −0.01 to 0.11), or 90-day mortality (RD, −0.01; 95% CI, −0.02 to 0.0). There was low certainty of evidence that RAMIE was associated with a longer disease-free survival compared with VAMIE. For OE comparisons (data not pooled), RAMIE was associated with a longer operative time, decreased EBL, and less pulmonary and total complications.

**Conclusions and Relevance:**

In this study, RAMIE had similar outcomes as VAMIE but was associated with fewer pulmonary complications compared with VAMIE and OE. Studies on long-term functional and cancer outcomes are needed.

## Key Points

**Question:**

Are there differences in clinical outcomes with use of robot-assisted minimally invasive esophagectomy (RAMIE) compared with video-assisted minimally invasive esophagectomy (VAMIE) and open esophagectomy (OE) for cancer?

**Findings:**

In this systematic review and meta-analysis of 21 articles including 9355 patients, RAMIE was associated with fewer pulmonary complications than VAMIE but had otherwise similar outcomes. Compared with OE, RAMIE was associated with increased operative time, decreased estimated blood loss, and fewer pulmonary and total complications.

**Meaning:**

In this study, RAMIE was associated with fewer procedural complications; further work is needed to explore surgical approach and long-term oncologic outcomes.

## Teaser

This systematic review and meta-analysis evaluates the literature for clinical outcomes of robot-assisted minimally invasive esophagectomy (RAMIE) compared with video-assisted minimally invasive esophagectomy (VAMIE) and open esophagectomy (OE).

## Introduction

Worldwide adoption of robot-assisted surgery continues to increase, particularly for cancer and thoracic operations.^1,2,3^ Esophageal cancer is the seventh most common cancer diagnosis in the world each year, with an estimated 604 000 new cases in 2020.^4^ Esophagectomy is an important component of esophageal cancer treatment, often combined with perioperative chemoradiation for advanced disease. It is performed using a variety of approaches, including open, conventional minimally invasive (thoracoscopic and laparoscopic), or robot-assisted techniques. In 2016, more than 1800 robotic esophagectomies were performed worldwide, a 9-fold increase from 2009.^5^

Open transthoracic esophagectomy (OE), traditionally the main surgical approach,^6,7^ is a complex operation with morbidity and mortality of nearly 50% and 5%, respectively.^8^ Minimally invasive approaches have emerged, with laparoscopic and thoracoscopic techniques offering advantages that include fewer postoperative complications and possibly improved long-term oncologic outcomes compared with open surgery.^9,10,11,12^ Robot-assisted minimally invasive esophagectomy (RAMIE) may offer additional advantages because of the 540° wrist articulation, 3-dimensional vision, and greater magnification.^13,14^ Despite the rapid adoption of RAMIE, questions about its utility compared with other minimally invasive approaches and OE remain, especially with regard to long-term oncologic outcomes. Individual studies and systematic reviews comparing RAMIE with other minimally invasive approaches and OE have been limited by 1 or more of the following: inclusion of cases and/or studies not specific to esophageal cancer^15,16^; inclusion of studies without comparator groups (ie, case series)^17^; and comparison of minimally invasive esophagectomy with OE without analyzing RAMIE and video-assisted minimally invasive esophagectomy (VAMIE) separately.^18,19^ Discerning differences between techniques is further complicated by the various techniques used for esophagectomy (Ivor-Lewis, McKeown, and transhiatal) and by the fact that the contribution of the robot to the abdominal or thoracic phase is not clear. We conducted a systematic review and meta-analysis to evaluate the literature for clinical outcomes of RAMIE compared with VAMIE and OE.

## Methods

This review builds on a report commissioned by the Department of Veterans Affairs (VA) on the clinical and long-term outcomes of robot-assisted esophagectomy for cancer.^20^ This systematic review is reported using the Preferred Reporting Items for Systematic Reviews and Meta-analyses (PRISMA) standards,^21^ and the a priori protocol was registered in PROSPERO (CRD42020198907).

### Literature Search

We searched for articles published in PubMed, Cochrane, Ovid Medline, and Embase databases from January 1, 2013, to May 6, 2020, using terms related to robotic surgery or esophagectomy or cancer (eMethods in the Supplement). Prior to 2013, RAMIE was not widely performed. Additionally, the Dutch Chemoradiotherapy for Oesophageal Cancer followed by Surgery Study, published in 2012, demonstrated improved oncologic outcomes with neoadjuvant treatment, which has now become common in practice.^22,23^ Therefore, we considered studies published prior to 2013 to be insufficiently relevant to modern practice.

### Study Selection and Data Collection

Title, abstract, and full-text screening were completed by 2 independent team members (M.A.M., R.S., M.J.D., P.A.T., M.D.G., and M.M.G.), and disagreements were reconciled through group discussion. The inclusion criteria required that studies compared RAMIE with VAMIE or OE and that both groups used the same or similar surgical approach (eg, Ivor-Lewis, McKeown, transthoracic, transhiatal). Observational studies from the same data source, either large databases or single institutional databases, were considered to have large overlap if more than 50% of the same patients were included in multiple studies or if there was a greater than 50% overlap in the enrollment period. In this instance, the study with the most recent data and the most outcomes of interest was included. Studies were excluded if there were fewer than 10 patients in either group, as smaller studies were considered to have a high risk of bias.

Data extraction was completed in duplicate (M.A.M. and M.J.D.; M.A.M. and M.D.G.; and M.A.M. and M.M.G.), and all discrepancies were resolved through group discussion. Data from 1 Chinese study was extracted by a member of the research team (M.M.G.) with assistance from a Chinese-American physician fluent in both languages who also has extensive experience in systematic reviews.^24^ Data were abstracted on study design and preoperative patient and tumor characteristics, intraoperative outcomes, short-term outcomes, and long-term clinical and oncologic outcomes. Short-term was defined as outcomes reported for as long as 90 postoperative days, and long-term was defined as outcomes after 90 days. Intraoperative outcomes of interest included operative time, estimated blood loss (EBL), and number of lymph nodes (LN) harvested. Of note, we used total operative time when it was reported. The short-term outcomes of interest were anastomotic leak, recurrent laryngeal nerve (RLN) palsy and/or hoarseness, pulmonary complications (ie, pneumonia, pleural effusion), hospital length of stay (LOS), total postoperative complications, and mortality within 90 days. For LOS, we elected to only plot US-based studies in the analysis figures because of known international variations in LOS.^25,26,27,28^ We reported total postoperative complications (or major complications) when available. Long-term oncologic outcomes of interest were cancer recurrence and disease-free survival (DFS).

### Risk of Bias and Certainty of Evidence

Randomized clinical trials (RCTs) were assessed for risk of bias using the Cochrane Risk of Bias tool.^29^ Observational studies were assessed for risk of bias with the Cochrane Risk of Bias In Nonrandomized Studies of Interventions (ROBINS-I) tool.^30^ We used the criteria of the Grading of Recommendations Assessment, Development, and Evaluation (GRADE) working group (ie, study limitations, consistency, directness, and precision) to assess overall certainty of the evidence.^31^

### Statistical Analysis

#### RAMIE vs VAMIE

##### Meta-analysis

There was an insufficient number of studies to support a meta-analysis of RCTs for comparing RAMIE with VAMIE or RAMIE with OE (1 RCT for each comparison). However, there were 9 well-designed observational studies comparing RAMIE with VAMIE that used propensity matching to approximate the equivalence of confounding variables between treatment group. Therefore, we conducted a meta-analysis comparing RAMIE with VAMIE using these matched studies. Pooled estimates for continuous variables (ie, EBL and LN harvest) were reported as mean difference (MD) with their 95% CIs. Pooled estimates for categorical variables (ie, anastomotic leak, RLN palsy, pulmonary/total complications, and mortality) were reported as risk difference (RD) with their 95% CIs. The DerSimonian and Laird random-effects model was used to account for the possible presence of heterogeneity.^32^ Unexplained heterogeneity was quantified by the *I*^2^ statistic.^33^ The presence of publication bias was evaluated using Begg rank correlation and Egger regression tests.^34,35^ Two-tailed *P* < .05 was considered statistically significant.

##### Nonpooled Data

While a meta-analysis was performed with the propensity-matched studies comparing RAMIE and VAMIE, a nonpooled analysis with the additional RCT and 5 unmatched observational studies was also performed for the RAMIE vs VAMIE comparison. The nonpooled data are presented immediately after the meta-analysis for each outcome.

Continuous outcomes were analyzed using the mean or median along with a measure of dispersion (ie, SD or IQR) to calculate the difference and 95% CI between groups. For binary outcomes, the number of patients with the outcome was collected, and an RD was derived with its 95% CI.

#### RAMIE vs OE

A narrative synthesis of evidence from 9 studies was conducted comparing RAMIE with OE using the methods described in the previous, nonpooled data section. All analyses were performed with R version 4.0.2 (R Project for Statistical Computing).^36^

## Results

### Study Screening

**Figure 1.**
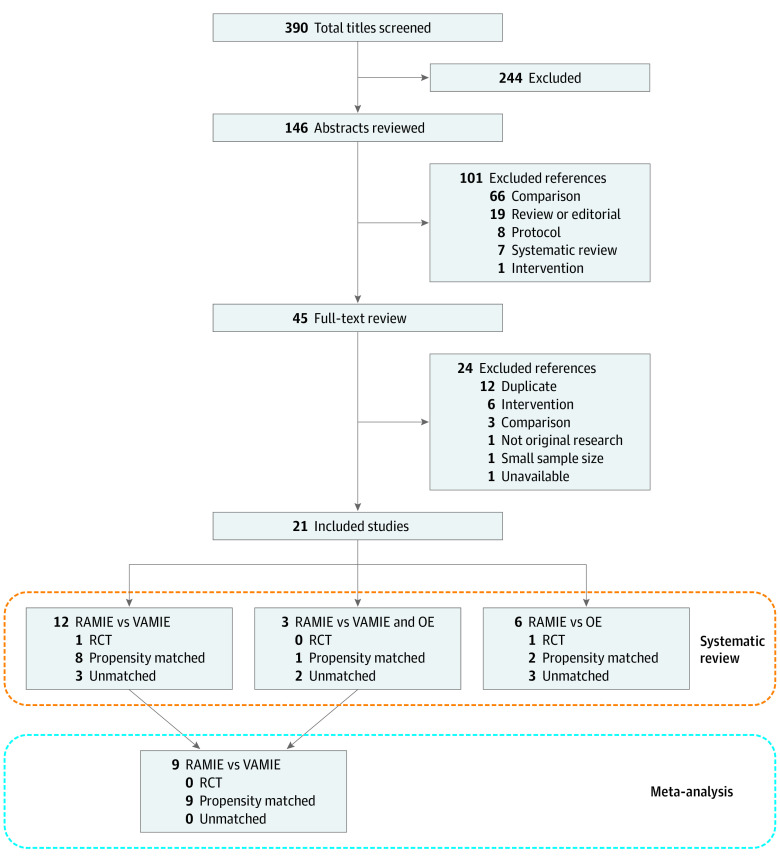
Flow Diagram of Literature Search and Selection OE indicates open esophagectomy; RAMIE, robot-assisted minimally invasive esophagectomy; RCT, randomized clinical trial; and VAMIE, video-assisted minimally invasive esophagectomy.

The study flow diagram is depicted in Figure 1. A total of 390 potentially relevant citations were identified, of which 146 were included at the abstract screening level. Six abstracts for systematic reviews^15,16,17,18,19,37^ and 1 abstract for a meta-analysis^38^ were identified at this stage. The meta-analysis of 8 observational studies^38^ compared RAMIE with VAMIE and found that RAMIE was associated with less EBL and vocal cord palsy. Two systematic reviews^15,16^ analyzed robotic foregut surgery and were not specific to esophageal cancer; 2 studies^18,19^ mostly compared minimally invasive esophagectomy (RAMIE and VAMIE combined) with OE; 1 systematic review^17^ included studies for robot-assisted gastrectomy and esophagectomy without comparator groups; and 1 systematic review^37^ did not specifically include RAMIE. From these, 45 studies underwent full-text review. Twenty-three full-text studies were excluded for various reasons, including wrong intervention, wrong comparison, and duplicate study (Figure 1). Ultimately, 21 studies including 9355 patients were identified as meeting inclusion criteria.^24,39,40,41,42,43,44,45,46,47,48,49,50,51,52,53,54,55,56,57,58^

### Study Characteristics

Characteristics of the studies, including operative technique and location of the anastomosis, are provided in eTable 1 and eTable 2 in the Supplement. Of the 21 studies that reported clinical outcomes, 2 were RCTs,^24,54^ and the remaining were observational studies. One RCT from China^24^ randomized 192 patients with esophageal squamous cell carcinoma to RAMIE (robot-assisted thoracic and abdominal portions) or total thoraco-laparoscopic VAMIE. The other RCT from the Netherlands^54^ randomized 109 patients with esophageal cancer to RAMIE (robot-assisted thoracic portion and laparotomy) or OE (thoracotomy and laparotomy).

Of the 19 observational studies, 11 were propensity matched for patient characteristics and factors, such as age, sex, body mass index, comorbidities, receipt of neoadjuvant treatment, and cancer staging (eTable 3 in the Supplement).^39,40,41,42,45,46,49,53,56,57,58^ Most were from East Asia (6 studies from China^40,41,43,45,56,58^; 3 studies from South Korea^46,51,57^; 1 study from Japan^50^; and 1 study from Taiwan^39^), with 5 studies originating from the United States.^42,47,49,52,55^ The RAMIE and non-RAMIE cohorts of each study had comparable surgical approaches and varied in size from 36 to 5553 patients.

Ten observational studies^39,40,41,45,48,49,51,53,56,58^ exclusively compared transthoracic RAMIE with VAMIE, and 4 observational studies^46,50,52,57^ exclusively compared transthoracic RAMIE with OE. One study^44^ used the robot for the abdominal portion only. Three studies^42,43,47^ compared VAMIE, RAMIE, and OE, and 1 study^55^ compared transhiatal VAMIE with transhiatal RAMIE. Two studies^42,49^ were from large national databases. The study from the National Surgical Quality Improvement Program database^49^ compared OE with all minimally invasive esophagectomies (RAMIE and VAMIE combined) for the primary analysis but performed a secondary analysis comparing VAMIE and RAMIE with 2:1 propensity matching. Only data from the secondary analysis were abstracted for this review. The other database study^42^ analyzed patients from the National Cancer Database and compared RAMIE, VAMIE, and OE.

### Publication and Risk of Bias Assessments

Publication bias was not detected for any outcomes investigated in the meta-analysis comparing RAMIE with VAMIE (eTable 4 in the Supplement). Publication bias could not be assessed for the narrative synthesis comparing RAMIE with OE. Bias in the measurement classification of interventions, bias due to deviation from intended interventions, and bias in selection of the reported result were generally low (eTable 5 in the Supplement).

### RAMIE vs VAMIE: Meta-analysis and Nonpooled Data

#### LN Harvest

**Figure 2.**
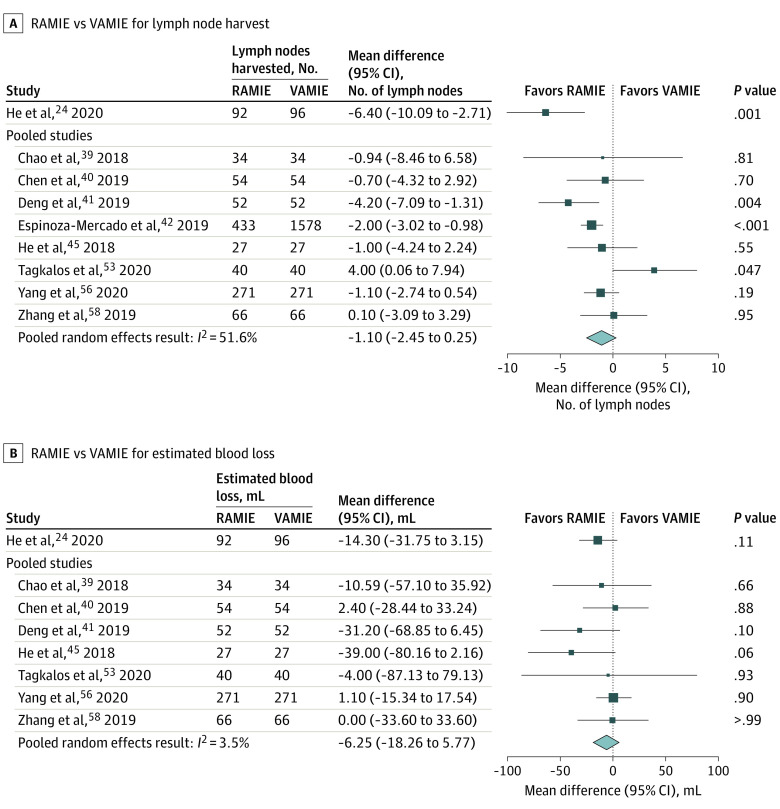
Forest Plots Comparing Robot-Assisted Minimally Invasive Esophagectomy (RAMIE) With Video-Assisted Minimally Invasive Esophagectomy (VAMIE) for Intraoperative Outcomes

Eight studies^39,40,41,42,45,53,56,58^ in the meta-analysis reported LN harvest. Two propensity-matched studies^41,42^ reported a larger LN harvest with RAMIE, and 1 matched study^53^ reported a larger harvest with VAMIE. The pooled random-effects result was an adjusted MD of −1.10 LNs (95% CI, −2.45 to 0.25 LNs; *I*^2^ = 51.6%). In comparison, the RCT^24^ demonstrated a significantly larger LN harvest with RAMIE (MD, −6.40 LNs; 95% CI, −10.09 to −2.71 LNs; *P* = .001). Figure 2A presents the results of the RCT by He et al^24^ graphically as well as the pooled analysis of the matched observational studies. The nonpooled unmatched observational studies had similar results as those included in the meta-analysis: 2 studies^47,51^ reported a larger LN harvest with RAMIE, and 3 studies^43,48,55^ reported no difference.

#### EBL

The 7 studies^39,40,41,45,53,56,58^ in the pooled analysis reporting outcomes for EBL found no difference between approaches. The pooled random-effects result was an adjusted MD of −6.25 mL (95% CI, −18.26 to 5.77 mL; *I*^2^ = 3.5%) (Figure 2B). Similarly, the RCT^24^ and 4 additional observational studies^43,47,48,51^ also found no difference in EBL between approaches.

#### Anastomotic Leak

**Figure 3.**
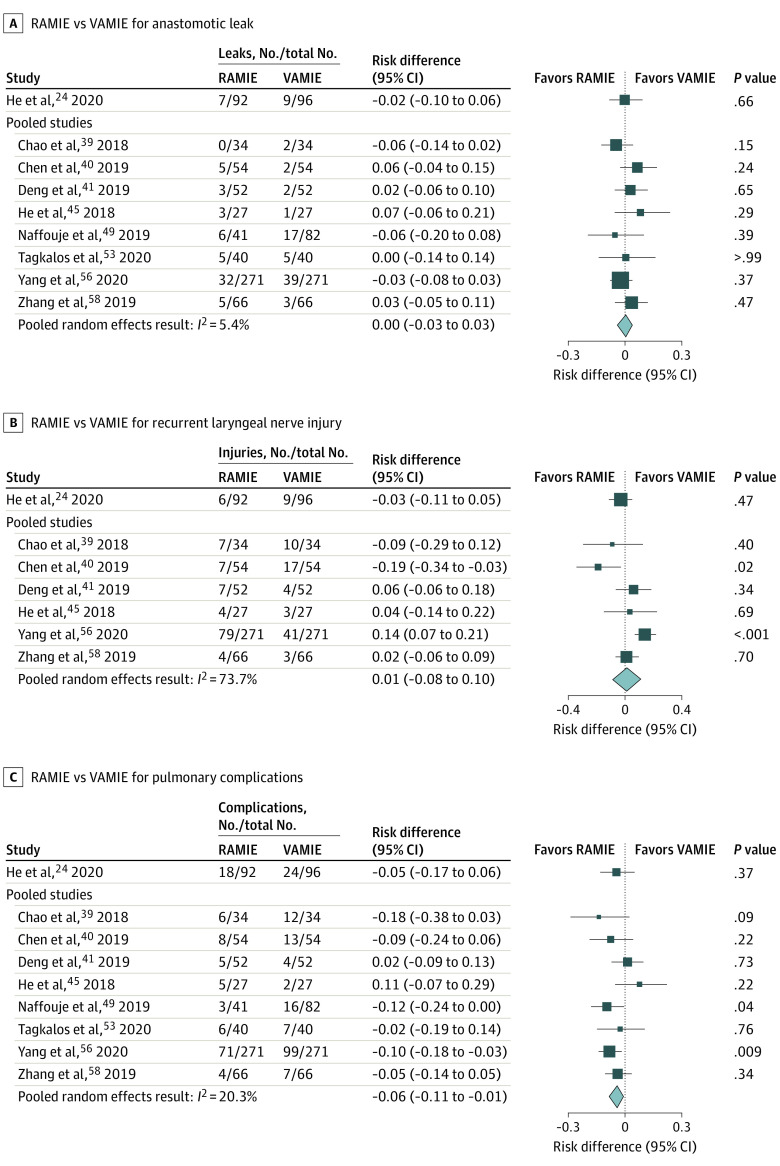
Forest Plots Comparing Robot-Assisted Minimally Invasive Esophagectomy (RAMIE) With Video-Assisted Minimally Invasive Esophagectomy (VAMIE) for Anastomotic Leak, Recurrent Laryngeal Nerve Injury, and Pulmonary Complications

Eight studies^39,40,41,45,49,53,56,58^ reporting data for anastomotic leak were included in the meta-analysis. Cervical anastomoses were performed in 5 studies (all McKeown).^39,40,41,45,56^ Three studies^49,53,58^ directly compared intrathoracic anastomoses with an Ivor-Lewis approach in both study groups. There was no difference in anastomotic leak rate in any of the studies, and there was no clear difference between RAMIE or VAMIE when evaluating studies with a cervical or intrathoracic anastomosis separately. The pooled random effects result was an adjusted RD of 0.0 (95% CI, −0.03 to 0.03; *I*^2^ = 5.4%) (Figure 3A).

The RCT^24^ and 5 unmatched observational studies^43,47,48,51,55^ similarly showed no difference between approaches. Of the additional studies, a cervical anastomosis was used in 4 studies (3 McKeown^24,43,51^; 1 transhiatal^55^). One study^47^ was from a large database and compared robot-assisted Ivor-Lewis with an unspecified transthoracic VAMIE, suggesting at least 1 study group had an intrathoracic anastomosis. Another study^48^ did not specify whether an intrathoracic or cervical anastomosis was performed.

#### RLN Injury

Six studies^39,40,41,45,56,58^ reported outcomes for RLN injury for the meta-analysis. Four studies^39,41,45,58^ found no evidence of a difference between RAMIE and VAMIE. One matched study^40^ demonstrated a lower rate of RLN palsy with RAMIE, while another^56^ demonstrated a lower rate with VAMIE. The pooled random-effects result was an adjusted RD of 0.01 (95% CI, −0.08 to 0.10; *I*^2^ = 73.7%) (Figure 3B). From the additional studies not included in the meta-analysis, the RCT^24^ and 2 unmatched studies^43,51^ reported no difference, and 1 study^48^ reported a lower rate of RLN palsy with RAMIE.

#### Pulmonary Complications

Eight studies^39,40,41,45,49,53,56,58^ reporting pulmonary complications were included in the meta-analysis. RAMIE was associated with a lower risk of pulmonary complications; the pooled random-effects result was an adjusted RD of −0.06 (95% CI, −0.11 to −0.01; *I*^2^ = 20.3%) for a number needed to treat (NNT) of 17 (Figure 3C). The RCT^24^ and additional unmatched studies^43,47,48,51^ found that most studies had point estimates at the null value or favoring RAMIE.

#### Total Complications

**Figure 4.**
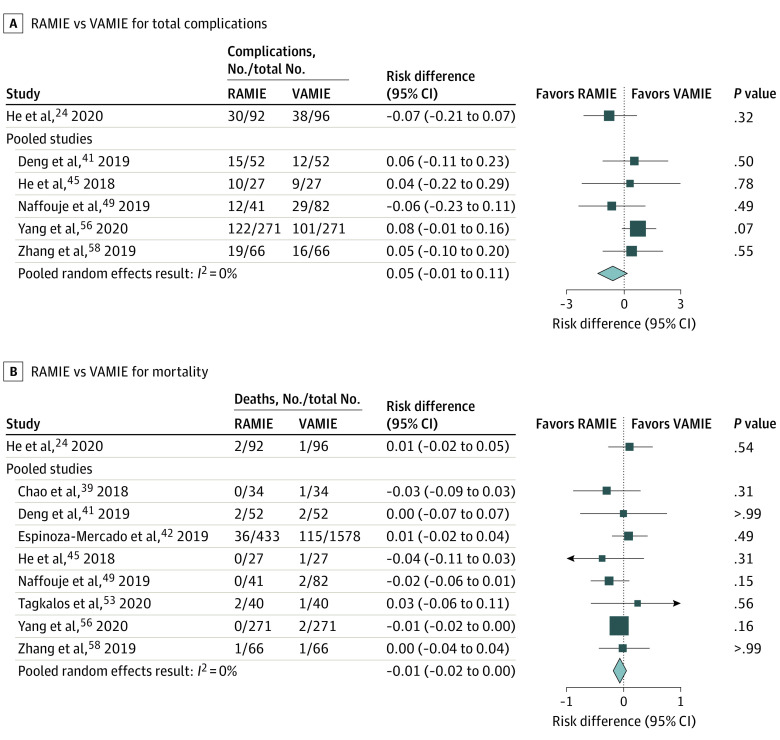
Forest Plots Comparing Robot-Assisted Minimally Invasive Esophagectomy (RAMIE) With Video-Assisted Minimally Invasive Esophagectomy (VAMIE) for Total Complications and 90-Day Mortality

Five studies^41,45,49,56,58^ reporting total complications were included in the meta-analysis. Pooled analysis did not reveal evidence of a difference for VAMIE compared with RAMIE (RD, 0.05; 95% CI, −0.01 to 0.11; *I*^2^ = 0.0%) (Figure 4A). The RCT^24^ and additional 4 unmatched observational studies^43,47,51,55^ also found no difference in total complications between approaches.

#### Mortality

Eight studies^24,39,41,42,45,49,53,56,58^ reporting mortality within 90 days were included in the meta-analysis. One propensity-matched study^40^ was excluded from the meta-analysis because both study arms had no deaths during the study period. One propensity-matched study^49^ reported 30-day mortality, and the remaining 7 studies^39,41,42,45,53,56,58^ reported 90-day mortality. The pooled random-effects result showed no difference between the approaches with an adjusted RD of −0.01 (95% CI, −0.02 to 0.0; *I*^2^ = 0.0%) (Figure 4B). The RCT^24^ and additional 4 observational studies^43,47,51,55^ similarly found no difference in mortality between study arms.

#### Operative Time

Fourteen studies^24,39,40,41,43,45,47,48,49,51,53,55,56,58^ reported operative time, of which 5 observational studies^41,45,47,53,58^ found shorter operative time with VAMIE. The RCT did not demonstrate a difference,^24^ and 1 matched study^56^ reported a shorter operative time with RAMIE (eFigure 1 in the Supplement). There was significant heterogeneity among the matched studies with nonoverlapping 95% CIs. Therefore, a pooled analysis was not performed for this outcome.

#### LOS

The 4 US observational studies^42,47,49,55^ assessing LOS found no significant difference between RAMIE and VAMIE (eFigure 2 in the Supplement). Nine non-US studies^24,39,40,41,43,45,53,56,58^ evaluated LOS, of which none demonstrated differences between RAMIE and VAMIE (eTable 1 in the Supplement). All but 1 of the non-US studies had a LOS with a mean or median greater than 10 days in both study groups,^58^ whereas all US studies had a mean or median of 10 days or less.

#### Long-term Oncologic Outcomes

Cancer recurrence was reported in the RCT^24^ and 1 propensity-matched observational study^56^ (eFigure 3 in the Supplement). With a median (IQR) follow-up time of 21 (3-57) months in the RCT,^24^ RAMIE and VAMIE had recurrence rates 14.9% (14 of 94) and 25.5% (25 of 98), respectively, that were not significantly different. It is unclear whether the recurrence rate was specific to locoregional, distant, or any recurrence. The observational study^56^ demonstrated no difference in any recurrence (ie, locoregional or distant) for RAMIE (11.8% [30 of 255]) and VAMIE (10.2% [26 of 254]) (*P* = .06). Locoregional recurrence was similar at 3.5% (9 of 255) for RAMIE and 3.9% (10 of 254) for VAMIE (*P* = .57). However, there were large differences in median (IQR) follow-up time between the two groups (RAMIE, 17.2 [1-33] months; VAMIE, 9.3 [1-33] months).^56^

DFS was reported in the RCT^24^ and 1 unmatched observational study^51^ (eFigure 3 in the Supplement). The median (IQR) time to recurrence in the RCT was 15 (9-42) months for RAMIE and 9 (3-42) months for VAMIE (*P* = .04). The observational study by Park et al^51^ reported a 5-year locoregional DFS of 88% (median follow-up, 17 months) for RAMIE and 74% (median follow-up, 26 months) for VAMIE (*P* = .10). One propensity-matched observational study by Yang et al^56^ found there was no difference in DFS between RAMIE and VAMIE in patients who had an R0 resection.^56^ A propensity-matched study by Espinoza-Mercado et al^42^ reported overall survival and found no difference between RAMIE (median, 58.8 [95% CI, 48-69] months) and VAMIE (median, 45.9 [95% CI, 33-58] months) (*P* = .60).^42^

### RAMIE vs OE: Narrative Synthesis

#### Intraoperative Outcomes

In the RCT,^54^ RAMIE had a longer mean (SD) operative time than OE (349 [56.9] minutes vs 296 [33.9] minutes; *P* < .001). Four of the observational studies^43,47,50,57^ reported a significant difference in operative time favoring OE, while 3 studies^44,46,52^ reported no difference. The number of LNs harvested was not different between RAMIE and OE in the RCT^54^ (27 LNs vs 25 LNs; *P* = .41),^54^ while 3 of the 7 observational studies^42,47,50^ reported an increase in LN harvest favoring RAMIE. Median (IQR) EBL was less for RAMIE (400 [258-581] mL) compared with OE (568 [428-800]; *P* < .001) in the RCT,^54^ which was supported by the observational studies, with 3 studies^43,46,57^ reporting no difference (eFigure 1 in the Supplement).

#### Short-term Postoperative Outcomes

Short-term postoperative outcomes are presented graphically in eFigure 2 in the Supplement. All studies that assessed anastomotic leak rate^43,44,46,47,50,52,54,57^ and RLN injury^43,46,50,52,54,57^ found no difference between RAMIE and OE. The rate of pulmonary complications was lower for RAMIE (32% [17 of 54]) compared with OE (58% [32 of 55]) in the RCT (relative risk, 0.54; 95% CI, 0.34-0.85; *P* = .005).^54^ Three observational studies^47,52,57^ also reported fewer pulmonary complications with RAMIE. The remaining 4 studies found no difference.^43,44,46,50^ Six studies^43,44,46,47,52,54^ evaluated total complications. The RCT,^54^ 1 propensity-matched study,^46^ and 1 unmatched observational study^44^ found a lower rate of total complications with RAMIE. The remaining studies^43,47,52^ found no difference. Mortality was assessed in 9 studies.^42,43,44,46,47,50,52,54,57^ One observational study^57^ demonstrated a higher mortality rate with OE compared with RAMIE; all remaining studies, including the RCT, found no difference.

#### Long-term Outcomes

The RCT^54^ and 1 propensity-matched observational study^57^ found no difference in any recurrence (locoregional or distant) or DFS between RAMIE and OE. Additionally, 1 propensity-matched observational study^42^ evaluating overall survival found no difference between the 2 approaches (eFigure 3 in the Supplement).

## Discussion

The findings of this study suggest that RAMIE is comparable with VAMIE in several respects while having some advantages compared with OE. The meta-analysis of intraoperative and short-term outcomes suggests a modest decrease in pulmonary complications associated with RAMIE compared with VAMIE, but there were no differences in EBL, LN harvest, anastomotic leak, RLN injury, total complications, or 90-day mortality. There was moderate certainty of evidence that there was no difference in LOS between RAMIE and VAMIE. There was low certainty of evidence that operative time was longer for RAMIE than VAMIE because of the heterogeneity of this outcome across studies. There was very low certainty of evidence that RAMIE may be associated with a longer DFS compared with VAMIE based on the RCT (Table).

**Table.** Certainty of Evidence

Outcome	Study limitations	Consistency	Directness	Precision	Certainty of evidence
**Intraoperative outcomes**					
Operating room time					
Greater for RAMIE than VAMIE	RCT: low; matched observational studies: moderate; unmatched observational studies: high	Inconsistent	Direct	Imprecise	Low
Greater for RAMIE than OE		Consistent	Direct	Precise	High
Lymph node harvest					
Greater for RAMIE than VAMIE	RCT: low; matched observational studies: moderate; unmatched observational studies: high	Inconsistent	Direct	Imprecise	Low
Greater for RAMIE than OE		Consistent	Direct	Imprecise	Moderate
Estimated blood loss					
Less for RAMIE than VAMIE	RCT: low; matched observational studies: moderate; unmatched observational studies: high	Consistent	Direct	Imprecise	Moderate
Less for than RAMIE than OE		Inconsistent	Direct	Precise	High
**Short-term postoperative outcomes**					
Anastomotic leak					
RAMIE equivalent to VAMIE	RCT: low; matched observational studies: moderate; unmatched observational studies: high	Consistent	Direct	Precise	High
RAMIE equivalent to OE		Consistent	Direct	Imprecise	Moderate
Recurrent laryngeal nerve injury					
RAMIE equivalent to VAMIE	RCT: low; matched observational studies: low; unmatched observational studies: moderate	Inconsistent	Direct	Precise	Low
RAMIE equivalent to OE		Consistent	Direct	Precise	Moderate
Pulmonary complications					
Fewer for RAMIE than VAMIE	RCT: low; matched observational studies: moderate; unmatched observational studies: high	Inconsistent	Direct	Precise	Moderate
Fewer for RAMIE than OE		Consistent	Direct	Imprecision	Moderate
Length of stay					
RAMIE equivalent to VAMIE	Matched observational studies: moderate; unmatched observational studies: high	Inconsistent	Direct	Imprecise	Moderate
Less for RAMIE than OE		Inconsistent	Direct	Imprecise	Very low
Total complications					
Greater for RAMIE than VAMIE	RCT: low; matched observational studies: moderate; unmatched observational studies: high	Consistent	Direct	Imprecise	Moderate
Fewer for RAMIE than OE		Consistent	Direct	Imprecise	Moderate
Mortality					
RAMIE equivalent to VAMIE	RCT: low; matched observational studies: moderate; unmatched observational studies: high	Consistent	Direct	Imprecise	High
RAMIE equivalent to OE		Inconsistent	Direct	Imprecise	Very low
**Long-term and oncologic outcomes**					
Recurrence					
Less for RAMIE than VAMIE	RCT: low; matched observational studies: moderate; unmatched observational studies: high	Inconsistent	Direct	Imprecise	Very low
RAMIE equivalent to OE		Inconsistent	Direct	Imprecise	Very low
Cancer-free survival					
Greater for RAMIE than VAMIE	RCT: low; matched observational studies: high; unmatched observational studies: high	Inconsistent	Direct	Imprecise	Very low
RAMIE equivalent to OE		Consistent	Direct	Imprecise	Very low

Abbreviations: OE, open esophagectomy; RAMIE, robot-assisted minimally invasive esophagectomy; RCT, randomized clinical trial; VAMIE, video-assisted minimally invasive esophagectomy.

Compared with OE, there was high certainty of evidence that RAMIE was associated with a lower EBL and a longer operative time. There was moderate certainty of evidence that RAMIE was associated with larger LN harvest and lower rates of pulmonary and total complications compared with OE. There was moderate certainty of evidence that there was no difference in anastomotic leak between RAMIE and OE. Finally, there was very low certainty of evidence that there are no differences in cancer recurrence or DFS between RAMIE and OE (Table).

A previous meta-analysis^38^ compared RAMIE with VAMIE based on a pooled analysis of 192 patients in each group from 8 case-controlled studies. Similar to our results, the authors found no differences in LN harvest, anastomotic leak, or postoperative mortality. However, they suggested RAMIE was associated with less EBL and a lower rate of RLN palsy, which was not observed in our larger analysis.

In our analysis, LOS was only evaluated in studies originating from the United States because of known international variations in hospital LOS practices.^25,27^ Most publications included in our study originated from China. A recent national study from 542 Chinese hospitals^26^ found that the mean LOS for 11 791 patients who underwent esophagectomy was 13.6 days. This is similar to what we observed after data extraction of the included studies. All US studies in our analysis had an LOS with a mean or median of 10 days or less, while all but 1 of the non-US studies had a mean or median greater than 10 days in both study groups.^58^

Additionally, regional variations of surgical practice and esophageal cancer epidemiology exist. The predominant histologic type of esophageal cancer in East Asian countries is squamous cell carcinoma, while adenocarcinoma predominates in the United States.^59,60,61^ Risk factors differ and underscore important clinical variation in patient populations and can potentially affect measured outcomes.^60,61^ Further, East Asian countries have a higher incidence of esophageal cancer and thus higher surgical volume.^59,60,61^

Several studies have demonstrated a potential benefit of minimally invasive esophagectomy and long-term oncologic outcomes. A systematic review and meta-analysis from 2019 comparing VAMIE with OE^12^ showed a significant survival advantage for VAMIE, with a 15% and 18% decrease in 3-year and 5-year mortality, respectively. However, this study did not separate RAMIE from VAMIE and primarily comprised observational studies. In our analysis, the paucity of included studies reporting long-term data hindered our ability to properly assess the long-term oncologic outcomes for RAMIE vs the comparator procedures.

Interestingly, the 5-year follow-up analysis of the Minimally Invasive vs Open Esophageal Resection trial^62^ found no difference in long-term oncologic outcomes between patients who underwent a hybrid esophagectomy (laparoscopy and thoracotomy) and open transthoracic esophagectomy. Instead, it found that major intraoperative and postoperative complications, as well as major pulmonary complications, negatively affected OS and DFS on multivariable analysis.^62^ In this study, we found that RAMIE was associated with a modest decrease in pulmonary complications compared with VAMIE. Accordingly, it is plausible that RAMIE may be associated with improved long-term oncologic outcomes.

### Limitations

This study has limitations. With the exception of 2 RCTs, most included studies were observational in design with limited data on long-term oncologic outcomes. This prevented us from drawing firm conclusions regarding the oncologic benefit of each surgical approach to esophagectomy. We used rigorous criteria to select observational studies, but these were all judged as being at high risk of bias. We used propensity-matched studies for the meta-analysis to help mitigate this bias, but there was still heterogeneity in the patient populations and even with the covariates used for matching. Comparisons across studies were also challenging because of inconsistent metrics for clinical outcomes. We acknowledge the possibility of publication bias; however, it is unlikely that we did not identify all RCTs in our screening process. Finally, our review represents only data that have been published, which is only a fraction of what could be known using the plethora of observational experiences from studies without comparison groups or that have not been published.

## Conclusions

In summary, RAMIE had similar outcomes to VAMIE and may be associated with fewer pulmonary complications. Compared with OE, RAMIE was associated with a longer operative time but fewer pulmonary and total complications. While the robot-assisted approach has the potential to provide beneficial outcomes, current data are too limited to offer definitive conclusions, particularly for long-term outcomes. Future research should include RCTs or well-designed prospective matched studies with adequate power and follow-up to assess long-term as well as oncologic outcomes for esophageal cancer, including determination of risks.

## Notes

Supplement. **eMethods.** Search Strategy **eTable 1. **Evidence Tables of Included Studies **eTable 2.** Operative Techniques of Included Studies **eTable 3.** Covariates for Propensity Matching in Studies Included in the Meta-analysis **eTable 4.** Publication Bias **eTable 5.** Risk of Bias Assessment **eFigure 1.** Forest Plot of Intraoperative Outcomes **eFigure 2.** Forest Plot of Short-term Outcomes **eFigure 3.** Forest Plot Long-term Outcomes
eReferences.
