# Supplementary material for: Comparison of Clinical Outcomes of Robot-Assisted, Video-Assisted, and Open Esophagectomy for Esophageal Cancer: A Systematic Review and Meta-analysis
Source: JAMA Netw Open. 2021 Nov 1;4(11):e2129228. doi: 10.1001/jamanetworkopen.2021.29228 (PMC8561331; doi:10.1001/jamanetworkopen.2021.29228)
Supplement: Supplement. — eMethods. Search Strategy eTable 1. Evidence Tables of Included Studies eTable 2. Operative Techniques of Included Studies eTable 3. Covariates for Propensity Matching in Studies Included in the Meta-analysis eTable 4. Publication Bias eTable 5. Risk of Bias Assessment eFigure 1. Forest Plot of Intraoperative Outcomes eFigure 2. Forest Plot of Short-term Outcomes eFigure 3. Forest Plot Long-term Outcomes eReferences. [file jamanetwopen-e2129228-s001.pdf]

Supplemental Online Content

Mederos MA, de Virgilio MJ, Shenoy R, et al. Comparison of clinical outcomes of robot-assisted, video-assisted, and open esophagectomy for esophageal cancer: a systematic review and meta-analysis. *JAMA Netw Open.* 2021;4(11):e2129228. doi:10.1001/jamanetworkopen.2021.29228

eMethods. Search Strategy

eTable 1. Evidence Tables of Included Studies

eTable 2. Operative Techniques of Included Studies

eTable 3. Covariates for Propensity Matching in Studies Included in the Meta-analysis

eTable 4. Publication Bias

eTable 5. Risk of Bias Assessment

eFigure 1. Forest Plot of Intraoperative Outcomes

eFigure 2. Forest Plot of Short-term Outcomes

eFigure 3. Forest Plot Long-term Outcomes

eReferences.

This supplemental material has been provided by the authors to give readers additional information about their work.

© 2021 Mederos MA et al. *JAMA Network Open.*

eMethods. Search Strategy

DATABASE SEARCHED &
TIME PERIOD COVERED:
PUBMED – 2013-2020
362 results

Search Strategy

(Randomized) OR ("control")) OR (randomly)) OR

(trial)) OR (comparative)) OR (prospective))AND

(Esophageal neoplasms[MESH terms]) OR ("Esophageal neoplasm")) OR ("Esophageal cancer")) OR ("Esophagus neoplasm")) OR ("Oesophageal neoplasm"))OR ("Oesophageal cancer")) OR ("Esophageal squamous cell carcinoma")) OR (Esophageal squamous cell carcinoma[MESH terms])) OR (Esophageal adenocarcinoma)) OR ("Esophagus cancer")))

AND

("minimally invasive") OR (Minimally-invasive)) OR (Laparoscopic)) OR (Thoracoscopic)) OR (Thoracolaparoscop*)) OR (Laparothoracoscop*)) OR (Video-assisted)) OR (video assisted)) OR (Video-assisted thoracic surgery)) OR (VATS)) OR (Open)) OR (Thoracotomy)) OR (Laparotomy)) OR (Transhiatal)) OR (McKeown)) OR ("Three-hole")) OR (3-hole)) OR (Ivor-Lewis)) OR (Esophagectomy)) OR (Oesophagectomy)) OR (Esophagectomies)) OR (Oesophagectomies)) OR (Esophageal resection)) OR (Oesophageal resection)) OR (Trans-hiatal)))

AND

"thoracic surgical procedures"[MESH Terms]) OR (Robotic Surgical Procedures [MeSH terms])) OR

(Robotics)) OR (Robot-assisted)) OR (Robot))Filters: from 2013 – 2020

DATABASE SEARCHED & TIME PERIOD COVERED:
OVID MEDLINE & Epub Ahead of Print, In-Process & Other Non-Indexed Citations and Daily
1 result

(randomized or "control" or randomly or

trial or comparative or prospective).af.

AND

exp Esophageal Neoplasms/ OR exp esophageal Squamous Cell Carcinoma/ OR

("esophageal neoplasm" or "esophageal cancer" or "esophagus neoplasm" or "oesophageal neoplasm" or "oesophageal cancer" or "esophageal squamous cell carcinoma" or "esophageal adenocarcinoma" or "esophagus cancer").mp. [mp=title, abstract, original title, name of substance word, subject heading word, floating sub-heading word, keyword heading word, organism supplementary concept word, protocol supplementary concept word, rare disease supplementaryconcept word, unique identifier, synonyms]

AND

("minimally invasive" or "minimally-invasive" or laparoscopic or thoracoscopic or thoracolaparoscop* or laparothoracoscop* or "video-assisted" or "video assisted" or "video-assisted thoracic surgery" or "VATS" or open or thoracotomy or laparotomy or transhiatal or McKeown or "three-hole" or "3-hole" or "Ivor-Lewis" or esphagectomy or oesophagectomy or esophagectomies or oesophagectomies or "esophageal resection" or "oesophageal resection" or

"trans- hiatal").mp. [mp=title, abstract, original title, name of substance word, subject heading word, floating sub-heading word, keyword heading word, organism supplementary concept word, protocol supplementary concept word, rare disease supplementary concept word, unique identifier, synonyms]

AND

exp Thoracic Surgical Procedures/ OR exp/Robotic Surgical Procedures/ OR (robotics or "robot-assisted" or robot).mp. [mp=title, abstract, original title, nameof substance word, subject heading word, floating sub-heading word, keyword heading word, organism supplementary concept word, protocol supplementary concept word, rare disease supplementary concept word, unique identifier, synonyms]

AND

Publication years 2013-2020

DATABASE SEARCHED & TIME PERIOD COVERED:
EMBASE – 2013-2020
15 results

randomized:ti,ab,kw OR control:ti,ab,kw OR randomly:ti,ab,kw OR trial:ti,ab,kw OR

comparative:ti,ab,kw OR prospective:ti,ab,kw AND

'esophageal neoplasms'/exp OR 'esophageal neoplasm':ti,ab,kw OR 'esophageal cancer':ti,ab,kw OR 'esophagus neoplasm':ti,ab,kw OR 'oesophageal neoplasm':ti,ab,kw OR 'oesophageal cancer':ti,ab,kw OR 'esophageal squamous cell carcinoma':ti,ab,kw OR 'esophageal squamous cell carcinoma'/exp OR 'esophageal adenocarcinoma':ti,ab,kw OR 'esophagus cancer':ti,ab,kw

AND

'minimally invasive':ti,ab,kw OR 'minimally-invasive':ti,ab,kw OR laparoscopic:ti,ab,kw OR thoracoscopic:ti,ab,kw OR thoracolaparoscop*:ti,ab,kw OR laparothoracoscop*:ti,ab,kw OR 'video-assisted':ti,ab,kw OR 'video assisted':ti,ab,kw OR 'video-assisted thoracic surgery':ti,ab,kw OR vats:ti,ab,kw OR open:ti,ab,kw OR thoracotomy:ti,ab,kw OR laparotomy:ti,ab,kw OR transhiatal:ti,ab,kw OR mckeown:ti,ab,kw OR 'three hole':ti,ab,kw OR '3-hole':ti,ab,kw OR 'ivor-lewis':ti,ab,kw OR esophagectomy:ti,ab,kw OR oesophagectomy:ti,ab,kw OR esophagectomies:ti,ab,kw OR oesophagectomies:ti,ab,kw OR 'esophageal resection':ti,ab,kw OR 'oesophageal resection':ti,ab,kw OR 'trans-hiatal':ti,ab,kw

AND

'thoracic surgicial procedures' OR 'robotic surgerical procedures' OR robotics:ti,ab,kw OR 'robot-

assisted':ti,ab,kw OR robot:ti,ab,kw AND

Publication years 2013-2020

DATABASE SEARCHED & TIME PERIOD COVERED:
COCHRANE Reviews – 2013- 2020
12 results

ID	Search Hits
#1	MeSH descriptor: [Esophageal Neoplasms] explode all trees
#2	MeSH descriptor: [Esophageal Squamous Cell Carcinoma] explode all trees
#3	(Randomized OR control OR randomly OR trial OR comparative OR prospective):ti,ab,kw
#4	("Esophageal neoplasm" OR "Esophageal cancer" OR "Esophagus neoplasm" OR "Oesophageal neoplasm" OR "Oesophageal cancer" OR "Esophagealsquamous cell carcinoma" OR "Esophageal adenocarcinoma" OR "Esophagus cancer"):ti,ab,kw

#5 #1 OR #2 OR #4

#6 ("minimally invasive" OR "Minimally-invasive" OR Laparoscopic OR Thoracoscopic OR Thoracolaparoscop* OR Laparothoracoscop* OR "Video- assisted" OR "video assisted" OR "Video-assisted thoracic surgery" OR VATS OR Open OR Thoracotomy OR Laparotomy OR Transhiatal OR McKeown OR "Three-hole" OR "3-hole" OR "Ivor-Lewis" OR Esophagectomy OR Oesophagectomy OR Esophagectomies OR Oesophagectomies OR"Esophageal resection" OR "Oesophageal resection" OR "Trans-hiatal"):ti,ab,kw

#7 MeSH descriptor:
[Thoracic Surgical Procedures]
explode all trees#8 MeSH
descriptor: [Robotic Surgical
Procedures] explode all trees

#9 (robotics OR "robot-assisted" OR
robot):ti,ab,kw (Word variations have been searched)#10
#7 OR #8 OR #9

#

1

1

#3 AND #5 AND #6 AND #10AND

Publication years Jan 2013- Dec2020

© 2021 Mederos MA et al. *JAMA Network Open.*

eTable 1. Evidence Tables of Included Studies

Patient Characteristics and intraoperative outcomes

**AuthorYear**<br>**Large Database (y/n)**<br>**Study Design**<br>**Propensity matching (y/n)**<br>**#Institutions/Surgeons**<br>**US (y/n)**<br>**VA (y/n)**	**Comparisons (e.g.,open vs robot Ivor-Lewis; VATSvs robot McKeown, etc.)**	**Patient & Tumor Characteristics Preop**<br>N<br>Age, mean yr (SD)Male, % Race/Ethnicity<br>NH-White, %NH-Black, %NH-Asian, %Hispanic, %<br>BMI, mean (SD)<br>ASA class, mean (SD) Comorbidity index (CCI):<br>Smoking current/former/unspecifiedDM<br>Albumin<br>Tumor Location (%)Stage<br>Neoadjuvant therapy (%)**Squamous (%) Adenocarcinoma (%)**			**Intraoperative Outcomes**<br>OR, time, min (SD)EBL, mL (SD)<br>Transfusions (%)Conversion (%)<br>Major Complications, N (%)<br>Lymph node harvest, N (std dev/IQR)Margins positive (%)		
		**Open**	**Robot**	**Other minimally-invasive approach**	**Open**	**Robot**	**Other minimally-invasive approach**

© 2021 Mederos MA et al. *JAMA Network Open.*

Chao, 2018[1]N RetrospectiveY Single institutionN N	McKeown (transthoracic robot + laparoscopic) vsMcKeown (VATS + laparoscopic). Stapled cervical anastomosis for both.		Matched N=34 Age: 56.76 (8.39) Male: 32 (94.1)BMI: NR ASA: NR Comorbidit yindex: 2.88 (1.27) Smoking: NRDM: NR Albumin: NR	Matched N=34 Age: 53.47 (8.69) Male: 33 (97.1)BMI: NR ASA: NR Comorbidity index: 2.88 (1.27) Smoking: NR DM: NR Albumin: NR		Matched Thoracic ORtime: 231.15 (42.84) EBL: 92.06 (99) Transfusions :3 (8.8) Conversions: 0 (0) LN harvest: 37.18 (18.25)	Matched Thoracic ORtime: 200.15 (103.48) EBL: 102.65 (96.67) Transfusions: 2 (5.9) Conversions: 0 (0) LN harvest: 36.24 (12.95)
			Tumor location:Upper: 10 (29.4) Mid: 15 (44.1) Lower: 9 (26.5) Stage: I/II: 16 (47.1) III: 18 (52.9) Neoadjuvan ttreatment: 17 (50) Squamous: 34 (100)	Tumor location: Upper: 10 (29.4 Mid: 19 (55.9) Lower: 5 (14.7) Stage: I/II: 16 (47.1) III: 18 (52.9) Neoadjuva nt treatment: 17 (50) Squamous: 34 (100)		Margins: R0: 34 (100)	Margins: R): 33 (97.1)

© 2021 Mederos MA et al. *JAMA Network Open.*

Chen, 2019[2]N<br>Retrospective<br>Y<br>1 surgical team/1 institution<br>N<br>N	Robotic McKeownvs MIE (laparoscopy & VATS) McKeown		MatchedN: 54<br>Age: 61.8 (9.4)<br>Male: 41 (75.9)<br>BMI: 22.7 (2.9)<br>ASA: NR<br>Comorbidityindex: NR<br>Smoking: 25 (46.3)<br>DM: 1 (1.9)<br>Albumin: NRcT stage:<br>1: 14 (25.9)<br>2: 7 (13)<br>3: 33 (61.1)<br>4a: 0<br>cN stage:<br>0: 30 (55.6)<br>1: 11 (20.4)<br>2: 11 (20.4)<br>3: 2 (3.7)<br>Neoadjuvant chemoradiation:14 (25.9)<br>Squamous cell carcinoma: 54 (100)	MatchedN: 54<br>Age: 61.8 (8.3)<br>Male: 43 (79.6)<br>BMI: 23 (2.7)<br>ASA: NR<br>Comorbidity index: NR<br>Smoking: 27 (50)<br>DM: 1 (1.9)<br>Albumin: NR<br>cT stage:<br>1: 15 (27.8)<br>2: 7 (13)<br>3: 31 (57.4)<br>4a: 1 (1.9)<br>cN stage:<br>0: 22 (40.7)<br>1: 14 (25.9)<br>2: 16 (29.6)<br>3: 2 (3.7)<br>Neoadjuvant chemoradiation:17 (31.5)<br>Squamous cell carcinoma: 54 (100)		Matched OR time: 187.2 (34)<br>EBL: 118.9 (77.4)<br>Conversion: NR<br>LN harvest: 25.4 (7.5)<br>Negative margins: 54 (100)	Matched OR time: 193.4 (27.1)<br>EBL: 116.5 (85.9)<br>Conversion: NRLN harvest: 24.7 (11.2)<br>Negative margins: 54 (100)

© 2021 Mederos MA et al. *JAMA Network Open.*

Deng, 2018[3]<br>N<br>Retrospective (prospective inclusion)Y<br>1 institution/2 surgeonsN<br>N	Robot McKeown (abd and thoracic portions) vs thoracolaparoscopic McKeown		Matched<br>N: 52<br>Age: 61 (7.2)<br>Male: 40 (76.9)<br>Height: 163.4 (6.8)<br>Weight: 58.7 (8)<br>ASA: NR<br>Major comorbidity: 12 (23.1)<br>Smoking: NR<br>DM: 3 (5.8)<br>Albumin: NR<br>Tumor location:<br>Upper: 10 (19.2)<br>Mid: 33 (63.5)<br>Lower: 9 (17.3)<br>Esophagogastric:0<br>Clinical Stage:I: 12 (23.1)<br>II: 36 (69.2)<br>III: 4 (7.7)<br>Squamous: 52 (100)	Matched<br>N: 52<br>Age: 60.9 (9.2)<br>Male: 39 (75)<br>Height: 163.5 (5.5)<br>Weight: 59.9 (8.5)<br>ASA: NR<br>Major comorbidity: 14 (26.9)<br>Smoking: NR<br>DM: 2 (3.8)<br>Albumin: NR<br>Tumor location:<br>Upper: 7 (13.5)<br>Mid: 30 (57.7)<br>Lower: 14 (26.9)<br>Esophagogastric:1 (1.9)<br>Clinical Stage:I: 9 (17.3)<br>II: 35 (67.3)<br>III: 8 (15.4)<br>Squamous: 52 (100)		Matched<br>OR time: 353 (71.8)<br>Thoracic time: 130.6 (28.7)<br>Abdominal time: 94.5 (21.6)<br>EBL: 96.3 (53.4)<br>LN harvest: 21.5 (8.4)<br>Mediastinal LN harvest: 11.8 (5.1)<br>Abdominal LN harvest: 9.7 (6.4)<br>R RLN LN harvest: 2.4 (1.9)<br>L RLN LN harvest: 1 (1.8)	Matched OR time: 274.2 (51.7)<br>Thoracic time: 121.7 (24.6)<br>Abdominal time: 87.5 (20.9)<br>EBL: 127.5 (127.8)<br>LN harvest: 17.3 (6.5)<br>Mediastinal LN harvest: 10.1 (4.3)<br>Abdominal LN harvest: 7.3 (5.1)R RLN LN harvest: 1.9 (2.2)L RLN LN harvest: 0.4 (0.8)
Espinoza-Mercado,2019[4]<br>Y (NCDB 2010-2015)<br>Restrospective<br>Y<br>1,500+<br>Y<br>N	Robot-assisted vs minimally invasive vs open	UnmatchedN: 3,542<br>Age (med, IQR): 63 (56-69)<br>Male: 2,995 (84.6)<br>White: 3,308 (93.4)<br>CCI zero: 2,434 (68.7)<br>CCI 1: 892 (25.2)<br>CCI ≥2: 216 (6.1)	Unmatched<br>N: 433<br>Age (med, IQR): 64 (57-70)<br>Male: 371 (85.7)<br>White: 398 (91.9)<br>CCI zero: 311 (71.8)<br>CCI 1: 95 (21.9)<br>CCI ≥2: 24 (5.9)<br>Tumor location:<br>Mid: 53 (12.2)<br>Lower: 380	Unmatched<br>N: 1,578<br>Age (med, IQR): 63 (57-69)<br>Male: 1,348 (85.4)<br>White: 1,490 (94.4)<br>CCI zero: 1,088 (68.9)<br>CCI 1: 384 (24.3)<br>CCI >2: 106	Margin: R0: 3,318 (94)<br>LN harvest(med, IQR): 13 (8-20)	Margin: R0: 408 (94.9)<br>LN harvest(med, IQR): 17 (11-24)	Margin: R0: 1,474 (94.1)<br>LN harvest(med, IQR): 15 (9-22)

© 2021 Mederos MA et al. *JAMA Network Open.*

		Tumor location: Mid: 422 (11.9) Lower: 3,120 (88.1) cT Stage: T1: 719 (20.5) T2: 761 (21.7) T3: 1,895 (54.1) cN stage: N0: 1,785 (50.8) N1: 1,329 (37.8) N2: 33 (9.5) Grade: Well-differentiated: 222 (7.1) Moderately-differentiated: 1,374 (43.9) Poorly-differentiated: 1,532 (49) pT stage: T1: 1,113 (35.8) T2: 633 (19.2) T3: 1,264 (40.6) pN stage: N0: 2,186 (64.4) N1: 734 (21.6) N2: 326 (9.6) p Stage: 0: 252 (7.1) 1: 1,140 (32.2) 2: 1,153 (32.6) 3: 997 (28.1) Neoadjuvant chemoradiation: 2,230 (63.6) Neoadjuvant chemotherapy:	(87.8) cT Stage: T1: 72 (16.7) T2: 79 (18.4) T3: 263 (61.2) cN stage: N0: 214 (49.4) N1: 171 (39.5) N2: 40 (9.2) Grade: Well-differentiated: 38 (9.7) Moderately-differentiated: 175 (44.6) Poorly-differentiated: 179 (45.7) pT stage: T1: 156 (37.9) T2: 83 (20.1) T3: 136 (33) pN stage: N0: 275 (64.9) N1: 99 (23.3) N2: 33 (7.8) p Stage: 0: 40 (10.1) 1: 143 (35.9) 2: 137 (34.4) 3: 78 (19.6) Neoadjuvant chemoradiation: 290 (67.1) Neoadjuvant chemotherapy:21 (4.9) Adenocarcinoma:	(6.8) Tumor location: Mid: 184 (11.7) Lower: 1,394 (88.3) cT Stage: T1: 346 (22.1) T2: 341 (21.8) T3: 826 (52.8) cN stage: N0: 821 (52.3) N1: 591 (37.6) N2: 133 (8.5) Grade: Well-differentiated: 145 (10.3) Moderately-differentiated: 593 (41.9) Poorly-differentiated: 676 (47.8) pT stage: T1: 569 (38.7) T2: 279 (19) T3: 511 (34.8) pN stage: N0: 987 (65.1) N1: 307 (20.3) N2: 163 (10.9) p Stage: 0: 123 (8.6) 1: 514 (36.1) 2: 475 (33.4) 3: 310 (21.8) Neoadjuvant chemoradiation: 981 (62.6) Neoadjuvant			

© 2021 Mederos MA et al. *JAMA Network Open.*

		215 (6.1)<br>Adenocarcinoma: 3,022 (85.3)<br>SCC: 520 (14.7)	363 (83.8)<br>SCC: 70 (16.2)	chemotherapy:89 (5.7)<br>Adenocarcinoma: 3,022 (85.3)<br>SCC: 520 (14.7)			
		Matched<br>N: 406<br>Age (med, IQR): 64 (56-68)<br>Male: 354 (87.2)<br>White: 382 (94.1)<br>CCI zero: 280 (69)<br>Tumor location: Lower: 346 (85.2)<br>Clinical Stage:0: 4 (1)<br>I: 113 (27.8)<br>II: 120 (29.6)<br>III: 169 (41.6)<br>cT Stage:<br>Tis: 4 (1)<br>T1: 93 (22.9)<br>T2: 87 (21.4)<br>T3: 211 (52)<br>T4: 8 (2)<br>cN stage: N0: 201 (51)<br>N1: 143 (35.2)<br>N2: 47 (11.6)<br>N3: 6 (1.5)<br>Grade:<br>Poorly-differentiated: 173 (42.6)<br>Neoadjuvant chemoradiation:	Matched<br>N: 406<br>Age: 64 (57-70)<br>White: 374 (92.1)<br>Male: 349 (86)<br>CCI zero: 296 (72.9)<br>Tumor location: Lower: 357 (87.9)<br>Clinical Stage:0: 6 (1.5)<br>I: 89 (21.9)<br>II: 138 (34)<br>III: 173 (42.6)<br>cT Stage:<br>Tis: 5 (1.2)<br>T1: 66 (16.3)<br>T2: 74 (18.2)<br>T3: 248 (61.1)<br>T4: 10 (2.5)<br>cN stage: N0: 207 (49.5)<br>N1: 160 (39.4)<br>N2: 38 (9.4)<br>N3: 7 (1.7)<br>Grade:<br>Poorly-differentiated: 172 (42.4)<br>Neoadjuvant chemoradiation: 276 (68)<br>Neoadjuvant	MIE vs RAMIE matched patient/pre-op characteristics not reported. The outcomes for matched are shown, however.	OR time: NR<br>EBL: NR<br>Conversion: NR<br>Margin:<br>R0: 374 (92.1)<br>LN harvest(med, IQR): 13 (7-21)	OR time: NR<br>EBL: NR<br>Conversion:NR<br>Margin:<br>R0: 383 (95)<br>LN harvest(med, IQR): 17 (11-24)	OR time: NR<br>EBL: NR<br>Conversion: NR<br>Margin:<br>R0: 388 (96.3)<br>LN harvest(med, IQR): 16 (10-22)

© 2021 Mederos MA et al. *JAMA Network Open.*

		252 (62.1)<br>Neoadjuvant chemotherapy: 18 (4.4)<br>Adenocarcinoma: 341 (84)	chemotherapy:17 (4.2)<br>Adenocarcinoma: 344 (84.7)				
Gong, 2020[5]<br>N<br>RetrospectiveN<br>1 institution/4 surgeons (only 2 performed robot)<br>N<br>N	Open vs total robotic vs thoracolaparoscopic McKeown	N: 77<br>Age: 59.77<br>Race: NR Male: 74 (96.1)BMI: NR<br>CCI:<br>0: 5 (6.5)<br>1: 28 (36.4)<br>2: 33 (42.9)<br>3: 11 (14.3)<br>4: 0<br>Smoking: NR<br>DM: NR<br>Albumin: NR<br>Tumor location:<br>Upper: 8 (10.39)<br>Mid: 37 (48.05)<br>Lower: 32 (41.56)<br>Clinical Stage:I: 2 (2.6)<br>II: 21 (27.27)<br>III: 47 (61.04)<br>IVA: 7 (9.09)<br>Neoadjuvant therapy: 40 (51.95)<br>Squamous cell carcinoma: 74 (96.1)<br>Adenocarcinoma: NR	N: 91<br>Age: 60.04<br>Race: NR<br>Male: 78 (85.71)<br>BMI: NR<br>CCI:<br>0: 8 (8.79)<br>1: 25 (27.47)<br>2: 40 (44)<br>3: 14 (15.38)<br>4: 4 (4.4)<br>Smoking: NR<br>DM: NR<br>Albumin: NR<br>Tumor location:<br>Upper: 7 (7.69)<br>Mid: 31 (34.07)<br>Lower: 53 (58.24)<br>Clinical Stage:I: 15 (16.48)<br>II: 38 (41.76)<br>III: 34 (37.36)<br>IVA: 4 (4.4)<br>Neoadjuvant therapy: 20 (21.98)<br>Squamous cell carcinoma: 86 (94.51)<br>Adenocarcinoma: NR	N: 144<br>Age: 60.22<br>Race: NR<br>Male: 130 (90.28)<br>BMI: NR<br>CCI:<br>0: 10 (6.94)<br>1: 44 (30.56)<br>2: 64 (4.44)<br>3: 22 (15.28)<br>4: 4 (2.78)<br>Smoking: NR<br>DM: NR<br>Albumin: NR<br>Tumor location:<br>Upper: 4 (2.78)<br>Mid: 72 (50)<br>Lower: 68 (47.22)<br>Clinical Stage:I: 20 (13.89)<br>II: 59 (40.97)<br>III: 47 (32.64)<br>IVA: 18 (12.5)<br>Neoadjuvant therapy: 28 (19.44)<br>Squamous cell carcinoma: 134 (93.06)<br>Adenocarcinoma: NR	OR time: 299.38 (57.98)<br>EBL: 289.61 (355)<br>Total LN harvest: 24.09 (10.77)<br>Cervical LN: 1.25 (4.3)<br>Upper mediastinum LN:4.33 (3.61)<br>Middle mediastinum LN: 7.81 (4.89)<br>Lower mediastinum: 1.77 (2.32)<br>Abdominal LN:8.94 (5.55)<br>Right RLN LN:2.14 (1.95)<br>Left RLN LN: 29 (37.66)<br>Margins positive:<br>R0 resection: 75 (97.4)	OR time: 318.02 (53.9)<br>EBL: 215.49 (125.4)<br>Total LN harvest: 22.84 (8.37)<br>Cervical LN: 0.29 (1.99)<br>Upper mediastinum LN: 6.22 (4.1)<br>Middle mediastinum LN: 6.34 (3.74)<br>Lower mediastinum: 1.9 (1.87)<br>AbdominalLN: 8.13 (5.53)<br>Right RLN LN: 2.74 (2.03) Left RLN LN: 2.35 (3.0)<br>Margins positive:<br>R0 resection:91 (100)	OR time: 321.13 (57.21)<br>EBL: 200.49 (59.54)<br>Total LN harvest: 23.07 (10.18)<br>Cervical LN:0.42 (1.7)<br>Upper mediastinum LN: 5.63 (3.88)<br>Middle mediastinum LN: 7.2 (4.69)<br>Lower mediastinum: 1.74 (2.18)<br>Abdominal LN: 8.1 (4.77)<br>Right RLN LN: 2.57 (2.08)<br>Left RLN LN: 1.95 (2.67)<br>Margins positive:<br>R0 resection:144 (100)

© 2021 Mederos MA et al. *JAMA Network Open.*

He, 2018[6]<br>N<br>RetrospectiveY<br>Single institutionN<br>N	McKeown RAMIE (abdominal and thoracic portions) vs VAMIE (MIE for thoracic and abdominal portions)		N: 27<br>Age: 61 (8)<br>Male: 20 (74.1)<br>BMI: 21.5 (2.7)<br>FEVI%: 94.6 (13.8)<br>CCI:<br>1: 1 (3.7)<br>2: 10 (37)<br>3: 13 (48.1)<br>4: 3 (11.1)<br>Tumor location:<br>Upper: 1 (3.7)<br>Mid: 18 (66.6)<br>Lower: 8 (29.6)pT stage:<br>T1: 4 (14.8)<br>T2: 13 (48.1)<br>T3: 10 (37)<br>pN stage: N0: 13 (48.1)<br>N1: 10 (37)<br>N2: 3 (11.1)<br>N3: 1 (3.7)<br>Tumor grade:<br>Well-differentiated: 2 (7.4)<br>Moderately differentiated: 19 (70.4)<br>Poorly differentiated: 6 (22.2)<br>Squamous: 23 (85.2)	N: 27<br>Age: 61.6 (9.8)<br>Male: 20 (74.1)<br>BMI: 21.9 (2.8)<br>FEVI%: 92.9 (23)<br>CCI:<br>1: 4 (14.8)<br>2: 8 (29.6)<br>3: 11 (40.7)<br>4: 4 (14.8)<br>Tumor location:<br>Upper: 3 (11.1)<br>Mid: 15 (55.6)<br>Lower: 9 (33.3)<br>pT stage: T1: 1 (3.7)<br>T2: 13 (48.1)<br>T3: 13 (48.1)<br>pN stage: N0: 18 (66.6)<br>N1: 8 (29.6)<br>N2: 1 (3.7)<br>N3: 0<br>Tumor grade:<br>Well-differentiated: 6 (22.2)<br>Moderately differentiated: 17 (63)<br>Poorly differentiated: 4 (14.8)<br>Squamous: 25 (92.6)		OR time: 349 (45)<br>EBL: 119 (72)<br>Lymph node harvest: 20 (7)	OR time: 285 (66)<br>EBL: 158 (82)<br>Lymph node harvest: 19 (5)

© 2021 Mederos MA et al. *JAMA Network Open.*

He, 2020[7]<br>N<br>RCT<br>N<br>1 institutions/NR surgeons<br>N<br>N	robot-assisted esophagectomy and thoraco-laparoscopic esophagectomy		N: 94<br>Age: 61.3 (8.2)<br>Race: NR<br>Male: 72%<br>BMI: 22.7<br>ASA:<br>1: 6 (6.4)<br>2: 82 (87.2)<br>3: 6 (6.4)<br>Smoking: NR<br>DM: 12 (12.8)<br>Tumor location: intrathoracic Upper: 9 (9.6)<br>Mid: 64 (68.1)<br>Lower: 21 (22.3)<br>Stage:<br>0-I: 51 (54)<br>II: 29 (30.9)<br>III: 14 (14.9)<br>Neoadjuvant: NR<br>Squamous: 94 (100)<br>Adenocarcinoma:0	N: 98<br>Age: 62.4 (9.1)<br>Race: NR<br>Male: 72%<br>BMI: NR<br>ASA: 22.8<br>1: 9 (9.2)<br>2: 80 (81.6)<br>3: 9 (9.2)<br>Smoking: NR<br>DM: 14 (14.3)<br>Tumor location: intrathoracic Upper: 7 (7.1)<br>Mid: 68 (69.4)<br>Lower: 23 (23.5)<br>Stage:<br>0-I: 49 (50.0)<br>II: 34 (34.7)<br>III: 15 (15.3)<br>Neoadjuvant: NR<br>Squamous: 98 (100)<br>Adenocarcinoma:0		Operating time:<br>Thoracic portion: NR<br>Abd + cervical: NR<br>Total: 304.2 (82.5)<br>Thoracic EBL: NS<br>Total EBL: 202.5 (73.4)<br>Transfusion: NR<br>Conversions total: 1<br>Thoracic conversion (to lap transhiatal): NR<br>Complications: NR<br>LN harvest: 22.2 (12.5)<br>Margins positive:<br>R0: 88 (95.7)	Operating time:<br>Thoracic portion: NR Abd + cervical:NR<br>Total: 315.5 (35.7)<br>Thoracic EBL:NS<br>Total EBL: 216.8 (44.6)<br>Transfusions:NR<br>Conversion total: 1<br>Thoracic conversion (to lap transhiatal):NR<br>Complications: NR<br>LN harvest: 20.1 (8.3)<br>Margins positive:<br>R0: 93 (96.9)
Jeong, 2016[8]N<br>RetrospectiveY<br>Single institutionN<br>N	Robot: 3 hole or 3 field (laparotomy; only throacic portion is robotic)<br><br>Open: Ivor-Lewis,3-hole, or 3-field	N: 159<br>Age >65 years: 50 (31%)<br>Male: 149 (94)<br>BMI: 22.7 (2.9)<br>ASA >2: 1 (0.6)<br>Smoking: 138 (87)<br>DM: 18 (11)<br>Albumin, med/IQR: 4.3 (4.1-4.5)	N: 88<br>Age >65 years: 25 (28%)<br>Male: 80 (91)<br>BMI: 22.6 (2.5)<br>ASA >2: 2 (2)<br>Smoking: 76 (86)<br>DM: 9 (10)<br>Albumin, med/IQR: 4.3 (4.2-4.6)<br>Tumor location:		OR time (hours, median/IQR): 4.4 (3.8-5.1)<br>EBL (med/IQR): 200 (150-300)<br>Intraop transfusion: 4<br>(2.5)<br>Intraop afib: 9 (6)	OR time (hours, median/IQR): 4.8 (3.9-5.6)<br>EBL (med/IQR): 200 (100-250)<br>Intraop transfusion: 0<br>Intraop afib: 7 (8)	

© 2021 Mederos MA et al. *JAMA Network Open.*

		Tumor location: NR<br>Clinical stage:I: 101 (64)<br>II: 46 (29)<br>III: 10 (6)<br>IV: 2 (1)	NR<br>Clinical stage:I: 59 (67)<br>II: 23 (26)<br>III: 5 (6)<br>IV: 1 (1)				
Meredith, 2019[9]N<br>Retrospective (propectively maintained database)N<br>Unkno wnY<br>N	Six approaches compared. The onlyrobotic approach is Ivor-Lewis. Comparable methods using otherapproaches in this study are open Ivor-Lewis and MIE transthoracic.	N: 475<br>Age: 64 (11)<br>Male: 412 (86.7)<br>BMI: 28 (6)ASA:<br>I: 2 (0.5)<br>II: 207 (54)<br>III: 172 (44.9)<br>IV: 2 (0.5)CCI: NR<br>Smoking: NRDM: NR<br>Albumin: NR<br>Tumor location: NR<br>Clinical Stage:I: 47 (12.6)<br>II: 142 (38.2)<br>III: 162 (43.5)<br>IV: 13 (3.5)<br>Neoadjuvant therapy: 274 (57.7)	N: 144<br>Age: 66 (10)<br>Male: 113 (78.5)<br>BMI: 28 (9)ASA:<br>I: 0<br>II: 50 (35.2)<br>III: 90 (63.4)<br>IV: 2 (1.4)CCI: NR<br>Smoking: NRDM: NR<br>Albumin: NR<br>Tumor location: NR<br>Clinical Stage:I: 32 (23.5)<br>II: 46 (33.8)<br>III: 56 (41.2)<br>IV: 1 (0.7)<br>Neoadjuvant therapy: 112 (77.8)	N: 95<br>Age: 62 (9)<br>Male: 81 (85.3)<br>BMI: 27 (5)<br>ASA:<br>I: 1 (1.1)<br>II: 53 (60.9)<br>III: 33 (37.9)<br>IV: 0 CCI: NR<br>Smoking: NR<br>DM: NR<br>Albumin: NR<br>Tumor location: NR<br>Clinical Stage:<br>I: 12 (14.3)<br>II: 24 (28.6)<br>III: 42 (50)<br>IV: 5 (6)<br>Neoadjuvant therapy: 73 (76.8)	OR time (min; mean/SD): 286 (69)<br>EBL: 289 (354)<br>Complications: 7 (1.5)<br>LN harvest: 10 (6)<br>Margins:<br>R1: 18 (3.8)<br>R2: 7 (1.5)	OR time (min; mean/SD): 409 (104)<br>EBL: 156 (107)<br>Complications :2 (1.4)<br>LN harvest: 20 (9)<br>Margi ns:R1: 0<br>R2: 0	OR time (min; mean/SD): 299 (87)<br>EBL: 189 (188)<br>Complications :2 (2.1)<br>LN harvest: 14 (7)<br>Margins:<br>R1: 6 (6.5)<br>R2: 0
Motoyama, 2019[10]N<br>Retrospective<br>N<br>1 institution<br>N<br>N	Robot: transthoracic (unclear how abdominal portion was performed)<br>MIE: transthoracic (unclear how abdominal portion was performed)		N: 21<br>Age (med/range):63 (44-76)<br>Male: 19 (90)BMI: NR ASA: NR CCI: NR<br>Smoking: NRDM: NR<br>Albumin: NR	N: 38<br>Age (med/range):66 (49-75)<br>Male: 32 (84)BMI: NR ASA: NR CCI: NR<br>Smoking: NR<br>DM: NR<br>Albumin: NR		OR time (min; med/range): 634 (529-699)<br>OR time thoracic: 320 (242-401)<br>EBL (med/range): 492 (195-1591)	OR time (min; med/range): 598.5 (475-761)<br>OR time thoracic: 312.5 (152-417)<br>EBL (med/range): 385 (177-3184)<br>EBL thoracic:

© 2021 Mederos MA et al. *JAMA Network Open.*

			Tumor location:<br>Upper: 6 (29)<br>Mid: 7 (33)<br>Lower: 8 (38)<br>Clinical T stage:<br>T1b: 5 (24)<br>T2: 5 (24)<br>T3: 11 (52)<br>Clinical N stage:N0: 8 (38)<br>N1: 10 (48)<br>N2: 3 (14)<br>Clinical stage:<br>IA: 4 (19)<br>IB: 3 (14)<br>IIA: 1 (5)<br>IIB: 3 (14)<br>IIIA: 7 (33)<br>IIIB: 3 (14)<br>Neoadjuvant Chemoradiation:12 (57)<br>Neoadjuvant chemo only: 0<br>Squamous cell carcinoma: 21 (100)	Tumor location:<br>Upper: 9 (24)<br>Mid: 16 (42)<br>Lower: 13 (34)<br>Clinical T stage:<br>T1b: 16 (42)<br>T2: 2 (5)<br>T3: 20 (53)<br>Clinical N stage:<br>N0: 19 (50)<br>N1: 13 (34)<br>N2: 6 (15)<br>Clinical stage:<br>IA: 14 (37)<br>IB: 2 (5)<br>IIA: 3 (8)<br>IIB: 2 (5)<br>IIIA: 11 (29)<br>IIIB: 6 (16)<br>Neoadjuvant Chemoradiation:19 (50)<br>Neoadjuvant chemo only: 1 (3)<br>Squamous cell carcinoma: 38 (100)		EBL thoracic: 110 (15-375)<br>LN harvest: 52 (36-104)<br>LN harvest mediastinal: 23 (11-41)	165 (23-559)<br>LN harvest: 59 (35-97)<br>LN harvest mediastinal: 20 (7-68)
Naffouje, 2019[11]<br>Y (NSQIP 2016-2017)<br>RetrospectiveY<br>Many<br>Y N	Open vs MIE (robot and all other MIE) Ivor-Lewis Secondary analysis compared laparoscopic vs robotic (2:1 propensity match)		MatchedN: 41<br>Age: 62.76 (9.98)<br>White: 39 (95.1)<br>Black: 1 (2.4)<br>Other race: 1 (2.4)<br>Male: 36 (87.8)<br>BMI: 27.8 (6.19)<br>ASA:<br>I: 0<br>II: 5 (12.2)	Matched<br>N: 82<br>Age: 63.27 (9.28)<br>White: 75 (91.5)<br>Black: 3 (3.7)<br>Other race: 4 (4.8)<br>Male: 72 (87.8)<br>BMI: 27.98 (5.6)<br>ASA:<br>I: 0<br>II: 11 (13.4)		OR time: 449 (116)<br>Conversion to open: 1 (2.4)<br>Negative margins: 35 (85.4)	OR time: 445 (96)<br>Conversion to open: 7 (8.5)<br>Negative margins: 74 (90.2)

© 2021 Mederos MA et al. *JAMA Network Open.*

			III: 35 (85.4)<br>IV: 1 (2.4)<br>Smoking: 12 (29.3)<br>DM: 6 (14.6)<br>Albumin: 3.83 (0.61)<br>cT stage: T1: 13 (31.7)<br>T2: 12 (29.3)<br>T3: 16 (39)<br>T4: 0<br>Tx: 0<br>cN stage: 0: 28 (68.3)<br>1: 8 (19.5)<br>2: 4 (9.8)<br>3: 1 (2.4)<br>Nx: 0<br>Neoadjuvant chemo: 30 (73.2)<br>Neoadjuvant radiation: 30 (73.2)<br>Adenocarcinoma:37 (90.2)<br>SCC: 4 (9.8)<br>Other malignancy: 0	III: 68 (82.9)<br>IV: 3 (3.7)<br>Smoking: 21 (25.6))<br>DM: 17 (20.7)<br>Albumin: 3.86 (0.38)<br>cT stage:<br>T1: 32 (39)<br>T2: 17 (20.7)<br>T3: 31 (37.8)<br>T4: 0<br>Tx: 2 (2.4)<br>cN stage: 0: 52 (63.4)<br>1: 13 (15.9)<br>2: 14 (17.1)<br>3: 0<br>Nx: 3 (3.7)<br>Neoadjuvant chemo: 62 (75.6)<br>Neoadjuvant radiation: 56 (68.3)<br>Adenocarcinoma: 76 (92.7)<br>SCC: 5 (6.1)<br>Other malignancy: 1 (1.2)			
Osaka, 2018[12]N<br>RetrospectiveN<br>1 institutionN<br>N	Robot (thoracic)with unknown method for abdomen vs. thoracotomy and unknown for abdomen	N: 30<br>Age (med, range): 63 (46-77)<br>Male: 27 (90)<br>BMI: NR<br>ASA: NR<br>CCI: NR<br>Smoking: NR<br>DM: NR	N: 30<br>Age (med, range): 62 (49-78)<br>Male: 27 (90)<br>BMI: NR ASA: NR CCI: NR<br>Smoking: NR<br>DM: NR		OR time, minutes (med, range): 398 (329-498)<br>EBL total (med, range): 388 (125-990)<br>EBL thoracic (med, range): 135 (44-325)<br>LN harvest (med,	OR time, minutes (med, range): 563 (476-713)<br>EBL total (med, range): 197 (10-640)<br>EBL thoracic (med, range): 21 (0-97)	

© 2021 Mederos MA et al. *JAMA Network Open.*

		Albumin: NR<br>Tumor location:<br>Upper: 1 (3.3)<br>Mid: 15 (50)<br>Lower: 14 (46.7)<br>Clinical Stage:I:<br>16 (53.3)<br>II: 13 (43.3)<br>III: 1 (3.3)<br>Neoadjuvant chemo: 8 (26.7)<br>Adenocarcinoma: NR<br>Squamous cell carcinoma: NR	Albumin: NR<br>Tumor location:<br>Upper: 1 (3.3)<br>Mid: 15 (50)<br>Lower: 14 (46.7)<br>Clinical Stage:I:<br>14 (46.7)<br>II: 10 (33.3)<br>III: 6 (20)<br>Neoadjuvant chemo: 13 (43.3)<br>Adenocarcinoma: NR<br>Squamous cell carcinoma: NR		range): 23 (12-39)	LN harvest (med, range): 25 (8-58)	
Park, 2016[13]N<br>RetrospectiveN<br>1 InstitutionN<br>N	Transthoracic robotvs transthoracic VATS. In the robotcohort, 90% were McKeown and 10% were Ivor-Lewis. Abdominal portion in the robotic cohortwas done robotically in 58%.<br>In the MIE cohort, abdominal portion was laparoscopic in 49%, 19% were Ivor-Lewis, and 81% were McKeown.		N: 62<br>Age: 64.3 (8)<br>Male: 57 (91.9)<br>BMI: 23.5 (2.8)ASA:<br>I: 21 (33.9)<br>II: 37 (59.7)<br>III: 4 (6.5)<br>Smoking: 49 (79)<br>Never smoker: 13 (21)<br>DM: 9 (14.5)<br>Albumin: NR<br>Tumor location:<br>Upper: 8 (12.9)<br>Mid: 15 (24.2)<br>Lower: 39 (62.9)<br>FEV1; pred%, SD: 101.6 (17.1)<br>Clinical stage:I:<br>23 (37.1)<br>II: 28 (45.2)<br>III: 11 (17.7)<br>Clinial T stage:T1:<br>31 (50)	N: 43<br>Age: 66.2 (7.4)<br>Male: 40 (93)<br>BMI: 23.3 (3.1)<br>ASA:<br>I: 11 (25.6)<br>II: 32 (74.4)<br>III: 0<br>Smoking: 35 (81.4)<br>DM: 11 (25.6)<br>Albumin: NR<br>Tumor location:<br>Upper: 7 (16.3)<br>Mid: 9 (20.9)<br>Lower: 27 (62.8)<br>FEV1; pred%, SD: 106.7 (13.8)<br>Clinical stage:I:<br>21 (48.8)<br>II: 15 (34.9)<br>III: 7 (16.3)<br>Clinial T stage:T1:<br>25 (58.1)<br>T2: 13 (30.2)		OR time:<br>Total: 490.3 (84)<br>Thoracic: 185.2 (67.4)<br>Abdominal: 305.1 (66.6)<br>EBL: 462.9 (493.9)<br>LN harvest: 37.3 (17.1)	OR time:<br>Total: 458.4 (111.9)<br>Thoracic: 120.1 (68.5)<br>Abdominal: 338.4 (105.4)<br>EBL: 466.8 (333)<br>LN harvest: 28.7 (11.8)

© 2021 Mederos MA et al. *JAMA Network Open.*

			T2: 21 (33.9)<br>T3: 10 (16.1)<br>Clinical N stage:N0: 42 (67.7)<br>N+: 20 (32.3)<br>Neoadjuvant chemoradiation:8 (12.9)<br>Squamous cell carcinoma: 62 (100)	T3: 5 (11.6)<br>Clinical N stage:<br>N0: 27 (64.3)<br>N+: 15 (35.7)<br>Neoadjuvant chemoradiation:4 (9.3)<br>Squamous cell carcinoma: 43 (100)			
Rolff, 2017[14]N<br>RetrospectiveN<br>1 InstitutionN<br>N	Open Ivor-Lewis vs Hybrid minimally-invasive Ivor-Lewis (Robot in abdomen + thoracotomy)	N: 160<br>Age (med, range): 65 (22-88)<br>Male: 125 (78)<br>BMI (med, range): 26.6 (15.6-43.7)<br>ASA:<br>1: 41 (26)<br>2: 80 (50)<br>3: 39 (24)<br>4: 0<br>CCI (med, range): 20.9 (0-100)<br>Smoking: NR<br>DM: NR<br>Albumin: NR<br>Tumor location: NR<br>Stage: NR<br>Neodjuvant therapy: NR<br>Adenocarcinoma: NR<br>Squamous cell carcinoma: NR	N: 56<br>Age (med, range): 66 (39-86)<br>Male: 50 (88)<br>BMI (med, range): 25.8 (18.8-31.2)<br>ASA:<br>1: 17 (30)<br>2: 28 (50)<br>3: 12 (21)<br>4: 1 (2)<br>CCI (med, range): 12.2 (0-100)<br>Smoking: NR<br>DM: NR<br>Albumin: NR<br>Tumor location: NR<br>Stage: NR<br>Neodjuvant therapy: NR<br>Adenocarcinoma: NR<br>Squamous cell carcinoma: NR		OR time (med, range): 248 (100-420)<br>EBL (med, range): 600 (100-4,400)<br>LN harvest (med, range): 23 (11-60)<br>Margins: NR	OR time (med, range): 232 (174-800)<br>EBL (med, range): 200 (50-1,970)<br>LN harvest (med, range): 28 (15-61)<br>Margins: NR	

Sarkaria, 2019[15]N<br>Non-randomized prospective trial N<br>Single institution/8 surgeons (2 performed robotic)<br>Y<br>N	Robotic Ivor Lewis (62/64) and McKeown (2/64) vsopen Ivor Lewis (103/106) Thoracoabdominal (3/106)<br><br>"All but 1 patientwho underwent MIE did so via a total RAMIE approach."	N: 106<br>Age (med, IQR): 63 (28-83)<br>Male: 91 (85.8)<br>BMI (med, IQR): 28.4 (16.9-49.5)<br>ASA:<br>II: 15 (14.2)<br>III: 84 (79.2)<br>IV: 7 (6.6)<br># of comorbidities:<br>0: 31 (29.2)<br>1-2: 62 (58.5)<br>>2: 13 (12.3)<br>Smoking: NR<br>DM: NR<br>Albumin: NR<br>Tumor location:<br>GE junction: 104 (98.1)<br>Distal: 2 (1.9)<br>Stage:<br>0: 2 (1.9)<br>I: 14 (13.2)<br>II: 26 (24.5)<br>III: 63 (59.4)<br>IV: 1 (0.9)<br>Neoadjuvant treatment: 87 (82.1)<br>Squamous: 7 (6.6)<br>Adenocarcinoma: 98 (92.5)<br>Other pathology:1 (0.9)	N: 64<br>Age (med, IQR): 61 (45-82)<br>Male: 53 (82.8)<br>BMI (med, IQR): 29.1 (15.6-47.8)<br>ASA:<br>II: 9 (14.1)<br>III: 51 (79.7)<br>IV: 4 (6.3)<br># of comorbidities:0: 23 (35.9)<br>1-2: 34 (53.1)<br>>2: 7 (10.9)<br>Smoking: NR<br>DM: NR<br>Albumin: NR<br>Tumor location:<br>GE junction: 60 (93.8)<br>Distal: 4 (6.3)Stage:<br>0: 1 (1.6)<br>I: 11 (17.5)<br>II: 17 (27)<br>III: 34 (54)<br>IV: 0 (0)<br>Neoadjuvant treatment: 48 (75)<br>Squamous: 4 (6.3)<br>Adenocarcinoma:59 (93.7)<br>Other pathology:0 (0)		OR time (hours, median & range): 5.44 (3.5-10.3)<br>EBL (med, range): 350 (100-2300)<br>LN harvest (med, range): 22 (0-50)<br>Margins positive (R1): 3 (2.8)	OR time (hours, median& range): 6.4 (4.9-10.6)<br>EBL (med, range): 250 (50-600)<br>LN harvest (med, range): 25 (14-56)<br>Margins positive (R1):2 (3.1)	

© 2021 Mederos MA et al. *JAMA Network Open.*

Tagkalos, 2019[16]N<br>Retrospective study of prospectively collected database<br>Y<br>1 institution/1 surgeonN<br>N	Robot (thoracic and abdominal) Ivor-Lewis vs. minimally-invasive(VATS and laparoscopy) Ivor-Lewis		Matched:<br>N: 40<br>Age: 62<br>BMI: 26.4ASA:<br>1-2: 22 (55)<br>3-4: 18 (45)<br>DM: 4 (10)<br>Pulmonary comorbidities: 8 (20)CV comorbidities: 15 (37.5)<br>Tumor location:<br>Upper: 0<br>Mid: 8 (20)<br>Lower: 32 (80)cT stage:<br>1-2: 7 (17.5)<br>3-4: 33 (82.5)<br>cN stage:0:<br>8 (20)<br>1: 32 (80)<br>Chemoradiation:22 (55)<br>Chemo only: 11 (27.5)	Matched:<br>N: 40<br>Age: 63<br>BMI: 25.6<br>ASA:<br>1-2: 19 (47.5)<br>3-4: 21 (52.5)<br>DM: 5 (12.5)<br>Pulmonary comorbidities: 6 (15)<br>CV comorbidities: 16 (40)<br>Tumor location:<br>Upper: 2 (5)<br>Mid: 6 (15)<br>Lower: 32 (80)<br>cT stage:<br>1-2: 10 (25)<br>3-4: 30 (75)<br>cN stage:0:<br>10 (25)<br>1: 30 (75)<br>Chemoradiation:21 (52.5)<br>Chemo only: 9 (22.5)		Matched OR time (med, range): 388 (255-475)<br>Abd time: 151 (80-250)<br>Thoracic time: 223 (170-320)<br>EBL: 339 (198)<br>LN harvest (median, range): 27 (13-84)<br>Negative margins: 38 (95)	Matched OR time (med, range): 321 (224-519)<br>Abd time: 125 (66-325)<br>Thoracic time: 201 (158-295)<br>EBL: 343 (181)<br>LN harvest (median, range): 23 (11-48)<br>Negative margins: 39 (97.5)
van der Sluis, 2019[17]N<br>RCT<br>N<br>1 institutions/2 surgeons<br>N<br>N	Open McKeown v Robot transthoracic with laparoscopic abdominal and open cervical portions	N: 55<br>Age: 65 (8.2)<br>Male: 42 (76)<br>BMI: 25.5 (4.7)<br>ASA:<br>1: 11 (20)<br>2: 34 (62)<br>3: 10 (18)<br>Comorbidity: 41 (75)<br>Smoking: NR<br>DM: NR	N: 54<br>Age: 64 (8.9)<br>Male: 46 (85)<br>BMI: 26.1 (4.4)ASA:<br>1: 13 (24)<br>2: 37 (69)<br>3: 6 (11)<br>Comorbidity: 43 (80)<br>Smoking: NR<br>DM: NR		Operating time:<br>Thoracic portion: 135 (23.3)<br>Abd + cervical:161 (30.1)<br>Total: 296 (33.9)<br>Thoracic EBL: 200 (195-313)<br>Total EBL: 568 (428-800)<br>Complications: 9 (16.4)	Operating time:<br>Thoracic portion: 170 (34.6)<br>Abd + cervical: 186 (38.7)<br>Total: 349 (56.9)<br>Thoracic EBL: 120 (78-200)	

© 2021 Mederos MA et al. *JAMA Network Open.*

		Albumin: NR<br>Tumor location:<br>Upper: 0 (0)<br>Mid: 8 (15)<br>Lower: 29 (53)<br>Clinical stage:<br>IA: 4 (7)<br>IIA: 3 (6)<br>IIB: 18 (33)<br>IIIA: 21 (38)<br>IIIB: 6 (11)<br>IIIC: 3 (6)<br>Neoadjuvant: 48 (87)<br>Squamous: 12 (23)<br>Adenocarcinoma: 43 (78)	Albumin: NR<br>Tumor location:<br>Upper: 1 (2)<br>Mid: 5 (9)<br>Lower: 26 (48)<br>Clinical stage: IA: 4 (7)<br>IIA: 5 (9)<br>IIB: 11 (20)<br>IIIA: 13 (24)<br>IIIB: 13 (24)<br>IIIC: 8 (15)<br>Neoadjuvant: 48 (90)<br>Squamous: 13 (24)<br>Adenocarcinoma:41 (76)		LN harvest: 25 (17-31)<br>Margins positive: R1: 2 (4)	Total EBL: 400 (258-581)<br>Conversion total: 3 (5.6)<br>Thoracic conversion (to lap transhiatal): 1 (1.9)<br>Complications:7 (13)<br>LN harvest: 27 (17-33)<br>Margins positive: R1: 2(4)	
Washington, 2019[18]N<br>RetrospectiveN<br>Single institution/1 surgeon<br>Y<br>N	Robotic vs laparoscopic transhiatal esophagectomy		N: 18<br>Age: 61.9 (range 42-76)<br>Male: 17 (94.4)<br>BMI: 27.6 (range 20.7-38.2)<br>ASA: NR<br>CCI: NR<br>Smoking: NR<br>DM: NR<br>Albumin: NR<br>Neoadjuvant treatment: 18 (100)<br>cT stage:1: 0<br>2: 4 (22.2)<br>3: 14 (77.8)<br>cN stage:0: 6 (33.3)<br>1: 12 (66.7)	N: 18<br>Age: 58.9 (range 40 to 70)<br>Male: 16 (88.9)<br>BMI: 27.5 (range 19.2-39.4)<br>ASA: NR<br>CCI: NR<br>Smoking: NR<br>DM: NR<br>Albumin: NR<br>Neoadjuvant treatment: 15 (83.3)<br>cT stage: 1: 3 (16.7)<br>2: 2 (11.1)<br>3: 12 (66.7)<br>cN stage:<br>0: 6 (33.3)<br>1: 8 (44.4)		OR time: 168 (24)<br>LN harvest: 14.28 (7.8)<br>Margins positive (R1):1 (5.6)	OR time: 164 (23.1)<br>LN harvest: 13.9 (8.5)<br>Margins positive (R1): 1 (5.6)

© 2021 Mederos MA et al. *JAMA Network Open.*

			Squamous: 4 (22.2)<br>Adenocarcinoma:14 (77.8)	Squamous: 3 (16.7)<br>Adenocarcinoma: 15 (83.3)			
Yang, 2019[19]<br>N<br>RetrospectiveY<br>Single institution/single surgeon<br>N<br>N	Robot McKeown (abd and thoracic portions) vs thoracolaparoscopic McKeown		MatchedN: 271<br>Age: 63.4 (7.1)<br>Male: 222 (81.9)<br>BMI: 23.2 (3)ASA:<br>I: 4 (1.5)<br>II: 243 (89.7)<br>III: 24 (8.9)CCI: NR<br>Smoking: NR<br>DM: NR<br>Albumin: NR<br>Tumor location:<br>Upper: 38 (14)<br>Mid: 169 (62.4)<br>Lower: 64 (23.6)<br>Clinical stage:I: 70 (25.8)<br>II: 97 (35.8)<br>III: 79 (29.2)<br>IV: 25 (9.2)<br>Neoadjuvant therapy: 29 (10.7)<br>Squamous cell:271 (100)	Matched<br>N: 271<br>Age: 63.5 (7.4)<br>Male: 221 (81.5)<br>BMI: 23.2 (2.9)<br>ASA:<br>I: 4 (1.5)<br>II: 242 (89.3)<br>III: 25 (9.2)CCI: NR<br>Smoking: NR<br>DM: NR<br>Albumin: NR<br>Tumor location:<br>Upper: 31 (11.4)<br>Mid: 171 (63.1)<br>Lower: 69 (25.5)<br>Clinical stage:I: 83 (30.6)<br>II: 86 (31.7)<br>III: 67 (24.7)<br>IV: 35 (12.9)<br>Neoadjuvant therapy: 28 (10.3)<br>Squamous cell:271 (100)		Matched OR time: 244.5 (60.4)<br>Thoracic time:85 (27.8)<br>EBL: 210.7 (86.8)<br>Thoracic conversion: 2 (0.7)<br>Total LN harvest: 20.3 (9.9)<br>Abdominal LN: 7.9 (4.8)<br>Thoracic LN: 12.4 (7)<br>RLN LN: 4.8 (3.3)<br>Negative margins: 255 (94.1)	Matched<br>OR time: 276 (59.4)<br>Thoracic time: 102.9 (28.6)<br>EBL: 209.6 (107.4)<br>Thoracic conversion: 16 (5.9)<br>Total LN harvest: 19.2 (9.6)<br>Abdominal LN: 6.8 (3.6)<br>Thoracic LN:12.4 (6.5)<br>RLN LN: 4.1 (3)<br>Negative margins: 254 (93.7)
Yun, 2019[20]N<br>Retrospective (prospective database)Y<br>1 surgeon/1 institution	Open (Ivor Lewis 54.4%; McKeown 45.6%) vs robot-assisted (Ivor Lewis 57.1%; McKeown 42.9%) (abdominal portion was either	Matched (Inverse probability of treatment weighting) N: 241<br>Age: 63 (7.8)	Matched (Inverse probability of treatment weighting) N: 130<br>Age: 63 (8.6)		Unadjusted<br>OR time: 240 (48.9)<br>EBL: 93.8 (140.9)<br>LN harvest: 38.3 (12.9)	Unadjusted<br>OR time: 275.6 (71.1)<br>EBL: 110.8 (125.8)<br>Conversion: 3 (2.3)	

© 2021 Mederos MA et al. *JAMA Network Open.*

N<br>N	robot-assisted or laparoscopic)	Male: 93%<br>BMI: 23.4 (2.8)<br>ASA: NR<br>CCI: NR<br>Smoking: 89.9%<br>DM: 14.2%<br>Albumin: NR<br>Tumor location:<br>Upper: 29.6%<br>Mid: 48.3%<br>Lower: 22.1%<br>Clinical Stage:I: 60.6%<br>II: 21.3%<br>III: 18.1%<br>Neoadjuvant treatment: 32.9%<br>Squamous cell carcinoma: 100%	Male: 92.6%<br>BMI: 23.4 (3.3)<br>ASA: NR CCI: NR<br>Smoking: 81.9%<br>DM: 14.4%<br>Albumin: NR<br>Tumor location:<br>Upper: 27.5%<br>Mid: 45.4%<br>Lower: 27.1%<br>Clinical Stage:I: 66.5%<br>II: 18.1%<br>III: 15.4%<br>Neoadjuvant treatment: 25.5%<br>Squamous cell carcinoma: 100%		Margins positive: 3.3%<br>R0: 233 (96.7)<br>R1: 7 (2.9)<br>R2: 1 (0.4)	LN harvest: 39.1 (13.8)<br>Margins positive: 2.3%<br>R0: 127 (97.7)<br>R1: 3 (2.3)<br>R2: 0	
Zhang, 2019[21]N<br>RetrospectiveY<br>1 surgeon/1 institutionN<br>N	Robot-assisted Ivor-Lewis (abdomen and thorax robot) vs thoracolaparoscopic Ivor-Lewis		MatchedN: 66<br>Age: 62.3 (7.8)<br>Male: 50 (75.8)<br>BMI: 22.9 (3.1)ASA:<br>1: 30 (45.5)<br>2: 33 (50)<br>3: 3 (4.5)<br>Comorbidity: 28 (42.2)<br>Smoking history:33 (50)<br>DM: NR<br>Albumin: NR<br>Tumor location:<br>Mid: 29 (43.9)<br>Lower: 37 (56.1)<br>Neoadjuvant therapy: 0<br>Adenocarcinoma:	Matched<br>N: 66<br>Age: 62 (7.8)<br>Male: 50 (75.8)<br>BMI: 23.1 (4.5)<br>ASA:<br>1: 26 (39.4)<br>2: 36 (54.5)<br>3: 4 (6.1)<br>Comorbidity: 32 (48.5)<br>Smoking history: 42 (63.6) DM: NR<br>Albumin: NR<br>Tumor location:<br>Mid: 26 (39.4)<br>Lower: 40 (60.6)<br>Neoadjuvant therapy: 0<br>Adenocarcinoma:		Matched<br>OR time: 302 (62.9)<br>EBL: 200 (100-262.5)<br>Conversion: 1 (1.5)<br>LN harvest: 19.2 (9.2)<br>Abd LN harvest: 8.9 (6.7)<br>Thoracic LN harvest: 10.3 (5.8)<br>R RLN LN harvest: 1.4 (1.6)<br>L RLN LN harvest: 1.3 (1.9)	Matched OR time: 274.7 (38)<br>EBL: 200 (150-245)<br>Conversion: 0<br>LN harvest: 19.3 (9.5)<br>Abd LN harvest: 7.3 (5.9)<br>Thoracic LN harvest: 11.9 (8.3)<br>R RLN LN harvest: 1.6 (2.8)L RLN LN harvest: 0.9 (1.9)<br>Margins positive: 0

			0<br>Squamous cell carcinoma: 64 (97)	0<br>Squamous cell carcinoma: 65 (98.5)		Margins positive: 0	

Short and long-term outcomes

**Author, Year**	**Comparisons (e.g., open vs robot Ivor-Lewis; VATSvs robot McKeown, etc.)**	**Short-Term Outcomes**<br>LOS, mean days (SD)<br>Readmissions, (%)<br>ICU stay<br>Pulmonary complications (pneumonia, pneumothorax, PE, ARDS, pleural effusion)<br>Chylothorax<br>MACE<br>Anastomotic leak, N (%)<br>Reoperations, N (%) RLN palsy<br>Mortality, N (%)			**Long-Term Outcomes**<br>Quality of life Overall survival<br>Cancer-specific survival<br>Follow up time		
		**Open**	**Robot**	**Other minimally-invasive approach**	**Open**	**Robot**	**Other minimally-invasive approach**
Chao, 2018[1]	McKeown (transthoracic robot + laparoscopic) vs McKeown (VATS + laparoscopic). Stapled cervical anastomosis forboth.		Matched LOS: 16.36 (5.79)<br>Readmissions: 5 (14.7)<br>ICU stay (hours): 31.85 (18.22)<br>Pneumonia: 2 (5.9)<br>Pleural effusion:4 (11.8)<br>30-day mortality: 0 (0)	Matched LOS: 17.82 (5.76)<br>Readmissions: 4 (11.8)<br>ICU stay (hours): 35.62 (47.33)<br>Pneumonia: 6 (17.6)<br>Pleural effusion: 6 (17.6)<br>30-day mortality: 0 (0)<br>90-day mortality: 1 (2.9)		NR	NR

© 2021 Mederos MA et al. *JAMA Network Open.*

			90-day mortality: 0 (0)<br>Anastomotic leak: 0 (0)<br>Reoperations: NR<br>RLN palsy: 7 (20.6)	Anastomotic leak:2 (5.9)<br>Reoperations: NR<br>RLN palsy: 10 (29.4)			
Chen, 2019[2]	Robotic McKeownvs MIE (laparoscopy & VATS) McKeown		Matched<br>LOS: 17.1 (10.1)<br>Readmissions: NR<br>ICU stay: 4 (6.3)<br>Pneumonia: 8 (14.8)<br>Chylothorax: 1 (1.9)<br>MACE: 2 (3.7)<br>Anastomotic leak: 5 (9.3)<br>Hoarseness/RLN palsy: 7 (13)<br>Mortality: 0 Total expense: $25,300 (9,000)<br>Expenses/day: $1,700 (700)	Matched<br>LOS: 15.2 (9.8)<br>Readmissions: NR<br>ICU stay: 2.5 (3.7)<br>Pneumonia: 13 (24.1)<br>Chylothorax: 2 (3.7)<br>MACE: 0<br>Anastomotic leak:2 (3.7)<br>Hoarseness/RLN palsy: 17 (31.5)<br>Mortality: 0<br>Total expense: $20,800 (9,000)<br>Expenses/day: $1,500 (400)		NR	NR
Deng, 2018[3]	Robot McKeown (abd and thoracic portions) vs thoracolaparoscopic McKeown		Matched<br>LOS: 14.3 (6.9)<br>Total major complications:15 (28.8)<br>Grade 1-2 complications: 9 (17.3)<br>Grade 3+ complications: 6 (11.5)<br>Pneumonia: 5 (9.6)	Matched LOS: 12.7 (7.7)<br>Total major complications: 12 (23.1)<br>Grade 1-2 complications: 6 (11.5)<br>Grade 3+ complications: 6 (11.5)<br>Pneumonia: 4 (7.7)<br>Chylothorax: 1		NR	NR

© 2021 Mederos MA et al. *JAMA Network Open.*

			Chylothorax: 0 Anastomotic leak: 3 (5.8) RLN palsy: 7 (13.5) In-hospital mortality: 1 (1.9) 90-day mortality: 2 (3.8)	(1.9) Anastomotic leak:2 (3.8) RLN palsy: 4 (7.7) In-hospital mortality: 2 (3.8) 90-day mortality: 2 (3.8)			
Espinoza-Mercado, 2019[4]	Robot-assisted vs minimally invasive vs open	Readmission:239 (6.9) LOS (med, IQR): 10 (8-15) 30-day mortality: 130 (3.7) 90-day mortality: 259 (7.4)	Readmission: 26 (6.1) LOS (med, IQR): 9 (7-14) 30-day mortality: 18 (4.2) 90-day mortality: 35 (8.2)	Readmission: 96 (6.2) LOS (med, IQR): 9 (8-14) 30-day mortality: 50 (3.2) 90-day mortality: 114 (7.3)	Overall survival (med, months; 95% CI): 43.6 (40-46)	Overall survival (med, months; 95% CI): 58.8 (47-69)	Overall survival (med, months; 95% CI): 47.5 (42-52)
		LOS (med, IQR): 10 (8-16) Readmission: 25 (6.2) ICU stay: NR Complications:NR 30-day mortality: 20 (4.9) 90-day mortality: 32 (7.9)	LOS (med, IQR): 9 (7-14) Readmission: 24 (6.1) ICU stay: NR Complications:NR 30-day mortality: 16 (3.9) 90-day mortality: 31 (7.6)	LOS (med, IQR): 9 (8-15) Readmission: 20 (4.9) ICU stay: NR Complications: NR 30-day mortality: 13 (3.2) 90-day mortality:25 (6.2)	Overall survival (med, months; 95% CI): 53.9 (42-85)	Overall survival (med, months; 95% CI): 58.8 (48-69)	Overall survival (med, months; 95% CI): 45.9 (33-58)
Gong, 2020[5]	Open vs total robotic vs thoracolaparoscopic McKeown	LOS: 16.66 (9.3) Reoperations:NR ICU stay: NR Total complications:	LOS: 16.57 (8.0) Reoperations:NR ICU stay: NR Total complications:	LOS: 18.73 (13.29) Reoperations: NR ICU stay: NR Total complications: 49 (34.03)	NR	NR	NR

© 2021 Mederos MA et al. *JAMA Network Open.*

		26 (33.77) Pneumonia: 10 (12.99) Atrial fibrillation: 10 (12.99) Anastomoticleak: 2 (2.6) Chylothorax: 3 (3.9) Bleeding: 0 RLN palsy: 12 (15.58) Wound infection: 2 (2.6)ICU readmission: 7 (9.09) Reoperations:NR Mortality (90-day): 2 (2.6)	33 (36.26) Pneumonia: 9 (9.89) Atrial fibrillation: 13 (14.29) Anastomoticleak: 4 (4.4) Chylothorax: 1 (1.1) Bleeding: 0 RLN palsy: 20 (21.98) Wound infection: 1 (1.67) ICU readmission: 6 (6.59) Reoperations:NR Mortality (90-day): 0	Pneumonia: 15 (10.42) Atrial fibrillation:21 (14.58) Anastomotic leak:10 (6.94) Chylothorax: 1 (0.7) Bleeding: 1 (0.7) RLN palsy: 34 (23.61) Wound infection: 0 ICU readmission: 12 (8.33) Reoperations: NR Mortality (90-day):0			
He, 2018[6]	McKeown RAMIE (abdominal and thoracic portions) vs VAMIE (MIE for thoracic and abdominal portions)		LOS: 13.8 (2) Overall complication rate: 10 (37) Pulmonary complications: 5 (18.5) Chylothorax: 0 Arrhythmia: 1 (3.7) Anastomotic leak: 3 (11.1) Bleeding: 1 (3.7) RLN palsy: 4 (14.8) 90-day mortality: 0	LOS: 12.8 (2.7) Overall complication rate:9 (33.3) Pulmonary complications: 2 (7.4) Chylothorax: 1 (3.7) Arrhythmia: 0 Anastomotic leak:1 (3.7) Bleeding: 1 (3.7) RLN palsy: 3 (11.1) 90-day mortality: 1 (3.7)		NR	NR

He, 2020[7]	robot-assisted esophagectomy and thoraco-laparoscopic esophagectomy		LOS (median):12 (5-78 range)<br>Readmissions: NR<br>ICU stay: 1.5 (1-24)<br>Pulmonary complications:18<br>Chylothorax: 2<br>MACE: NR<br>Mortality: 2<br>Anastomic leak:7<br>All complications: 30 (32.6)<br>Complications: Grade ≥2 directly relatedto surgery: NRGrade >2 overall: NR<br>Reoperations: NR<br>RLN palsy: 6	LOS (median): 13 (8-125) range<br>Readmissions: NR<br>ICU stay: 1.5 (1-20)<br>Pulmonary complications: 24<br>Chylothorax: 2<br>MACE: NR<br>Mortality: 1<br>Anastomotic leak:9<br>All complications:38 (39.6)<br>Complications: Grade ≥2 directly related to surgery: NR<br>Grade ≥2 overall:NR<br>Reoperations: NR<br>RLN palsy: 9		Overall survival: NR<br>Recurrence: 14<br>Recurrence free:1 yr: 92.4<br>3 yr: 87.3<br>Median recurrence time:15 (9-42)	Overall survival: NR<br>Recurrence: 25<br>Recurrence free:1 yr: 81.7<br>3 yr: 67.9<br>Median recurrence time:9 (3-42)
Jeong, 2016[8]	Robot: 3 hole or 3 field (laparotomy; only throacic portion is robotic)<br><br>Open: Ivor-Lewis,3-hole, or 3-field	LOS (med/IQR): 13 (12-16)<br>ICU stay (hours; med/IQR): 1.9 (1.8-2)<br>Complications(at least 1): 56 (35)<br>Pneumonia: 11 (7)<br>Anastomoticleak: 3 (2)<br>Afib: 9 (6)Vocal cord	LOS (med/IQR): 12 (10-15)<br>ICU stay (hours; med/IQR): 1.8 (1.8-1.9)<br>Complications(at least 1): 14 (16)<br>Pneumonia: 3 (3.4)<br>Anastomoticleak: 1 (1.1)<br>Afib: 2 (2.3)<br>Vocal cord		NR	NR	

		palsy: 1 (0.6)<br>Death: 1 (0.6)	palsy: 1 (1.1)<br>Death: 1 (1.1)				
Meredith, 2019[9]	Six approaches compared. The only robotic approach is Ivor-Lewis. Comparable methods using otherapproaches in this study are open Ivor-Lewis and MIE transthoracic.	LOS (med/range): 10 (1-115)<br>Complication rate: 145 (30.5)<br>Pulmonary complication: 81 (17.1)<br>Pneumonia: 72 (15.2)<br>PE: 9 (1.9)<br>Chylothorax: 5 (1.1)<br>MI: 6 (1.3)<br>Arrhythmia: 55 (11.6)<br>Anastomotic leak: 23 (4.8)<br>Reoperation: 12 (2.5)<br>90-day mortality: 7 (1.5)	LOS (med/range): 10 (4-66)<br>Complication rate: 34 (23.6)<br>Pulmonary complication: 14 (9.7)<br>Pneumonia: 10 (6.9)<br>PE: 3 (3.2)<br>Chylothorax: 1 (0.7)<br>MI: 1 (0.7)<br>Arrhythmia: 25 (17.4)<br>Anastomoticleak: 4 (2.8)<br>Reoperation: 0<br>90-day mortality: 2 (1.4)	LOS (med/range):9 (6-60)<br>Complication rate:28 (29.5)<br>Pulmonary complication: 18 (18.9)<br>Pneumonia: 8 (8.4)<br>Chylothorax: 1 (1.1)<br>MI: 3 (3.2)<br>Arrhythmia: 17 (17.9)<br>Anastomotic leak:4 (4.2)<br>Reoperation: 2 (2.1)<br>90-day mortality: 2 (2.1)		NR	NR
Motoyama, 2019[10]	Robot: transthoracic (unclear how abdominal portion was performed)<br><br>MIE: transthoracic (unclear how abdominal portionwas performed)		Chylothorax: 1 (5)<br>Pneumonia: 0<br>Anastomotic leak: 1 (5) Right RLN palsy: 2 (10)<br>Left RLN palsy:5 (24)	Chylothorax: 1 (3)<br>Pneumonia: 0<br>Anastomotic leak:3 (8)<br>Right RLN palsy:12 (32)<br>Left RLN palsy: 18 (47)		NR	NR

© 2021 Mederos MA et al. *JAMA Network Open.*

Naffouje, 2019[11]	Open vs MIE (robot and all other MIE) Ivor-Lewis Secondary analysis compared laparoscopic vs robotic (2:1 propensity match)		LOS (median, IQR): 7 (7-9.5) Readmissions: 6 (14.6) Pneumonia: 3 (7.3) PE: 1 (2.4) Transfusion: 1 (2.4) Reintubation: 4 (9.8) Superficial SSI:0 Deep SSI: 0 Organ space SSI: 3 (7.3) Overall complications (patients with at least one complication): 12 (29.3) Mortality: 0 Anastomotic leak: 6 (14.6) Reoperation: 5 (12.2)	LOS (median, IQR): 8 (7-12.25) Readmissions: 12 (14.6) Pneumonia: 16 (19.5) PE: 2 (2.4) Transfusion: 2 (2.4) Reintubation: 9 (11) Superficial SSI: 2 (2.4) Deep SSI: 1 (1.2) Organ space SSI:14 (17.1) Overall complications (patients with at least one complication): 28 (34.6) Mortality: 2 (2.4) Anastomotic leak:17 (20.7) Reoperation: 15 (18.3)			
Osaka, 2018[12]	Robot (thoracic)with unknown method for abdomen vs. thoracotomy and unknown for abdomen	LOS (med, range): 30 (22-35) Pulmonary complications: 3 (10) Anastomoticleak: 6 (20) SSI: 3 (10) Vocal cord palsy: 5 (16.7)	LOS (med, range): 17 (10-38) Pulmonary complications: 2 (6.7) Anastomoticleak: 3 (10) SSI: 0 Vocal cord palsy: 5 (16.7)		NR	NR	

© 2021 Mederos MA et al. *JAMA Network Open.*

Park, 2016[13]	Transthoracic robotvs transthoracic VATS. In the robotcohort, 90% were McKeown and 10% were Ivor-Lewis. Abdominal portion in the robotic cohortwas done robotically in 58%. In the MIE cohort, abdominal portion was laparoscopic in 49%, 19% were Ivor-Lewis, and 81% were McKeown.		LOS: NR<br>Readmissions:NR<br>ICU stay: NR<br>Pulmonary complication: 9 (14.5)<br>Anastomoticleak: 5 (8.1)<br>RLN palsy: 8 (12.9)<br>Complication ≥ Clavien Dindo IIIa: 10 (16.1)<br>30-day mortality: 1 (1.6)	LOS: NR<br>Readmissions: NR<br>ICU stay: NR<br>Pulmonary complication: 6 (14)<br>Anastomotic leak:1 (2.3)<br>RLN palsy: 10 (23.8)<br>Complication ≥ Clavien Dindo IIIa:9 (20.9)<br>30-day mortality: 0		Median follow-up: 17 months 5-year survival: 69%<br>5-year freedom of locoregional recurrence: 88%<br>5-year freedom of distal recurrence: 72%	Median follow-up: 26 months 5-year survival: 59%<br>5-year freedom of locoregional recurrence: 74%<br>5-year freedom of distal recurrence: 71%
Rolff, 2017[14]	Open Ivor-Lewis vs Hybrid minimally-invasive Ivor-Lewis (Robot in abdomen + thoracotomy)	LOS (med, range): 11.5 (8-101)<br>Complications (Clavien-Dindo):<br>≥1: 122 (76)<br>≥2: 91 (57)<br>≥3: 51 (32)<br>Pulmonary complications:81 (51)<br>Anastomotic leak: 11 (7)<br>30-day mortality: 3 (2)<br>90-day mortality: 5 (3)	LOS (med, range): 10 (8-69)<br>Complications (Clavien- Dindo):<br>≥1: 37 (65)<br>≥2: 22 (39)<br>≥3: 14 (25)<br>Pulmonary complications:24 (43)<br>Anastomotic leak: 4 (7)<br>30-day mortality: 0<br>90-day mortality: 3 (5)		NR	NR	

© 2021 Mederos MA et al. *JAMA Network Open.*

Sarkaria, 2019[15]	Robotic Ivor Lewis (62/64) and McKeown (2/64) vs open Ivor Lewis (103/106) Thoracoabdominal (3/106)<br><br>"All but 1 patient who underwent MIE did so via a total RAMIE approach."	Readmissions:17 (16)<br>LOS (med, range): 11 (6-131)<br>ICU admission:19 (19.8)<br>Complication (≥ grade 3): 55 (51.9)<br>Pulmonary complication: 36 (34)<br>Chylothorax: 1 (0.9)<br>MACE (afib): 2 (1.9)<br>Infection (any):38 (35.8)<br>Anastomotic leak: 10 (9.4)<br>RLN palsy: 0 (0)<br>30-day mortality: 2 (1.9)<br>90-day mortality: 4 (3.8)	Readmissions:13 (20.4)<br>LOS (med, range): 9 (5-17)<br>ICU admission:5 (7.8)<br>Complication (≥ grade 3): 25 (39.1)<br>Pulmonary complication: 9 (14.1)<br>Chylothorax: 0 (0)<br>MACE (afib): 1 (1.6)<br>Infection (any):11 (17.2)<br>Anastomoticleak: 2 (3.1)<br>RLN palsy: 2 (3.1)<br>30-day mortality: 1 (1.6)<br>90-day mortality: 1 (1.6)		Functional Assessment of Cancer Therapy–Esophageal (FACT-E): no difference between surgical approach	Functional Assessment of Cancer Therapy–Esophageal (FACT-E): no difference between surgicalapproach	
Tagkalos, 2019[16]	Robot (thoracic and abdominal) Ivor-Lewis vs. minimally-invasive(VATS and laparoscopy) Ivor-Lewis		Matched LOS (med, range): 12 (7-59)<br>ICU stay (med, range): 1 (1-43)<br>Pneumonia: 6 (15)<br>Anastomotic leak: 5 (12.5)<br>Wound infection: 0<br>30-day	Matched LOS (med, range): 12.5 (9-54)<br>ICU stay (med,range): 2 (1-17)<br>Pneumonia: 7 (17.5)<br>Anastomotic leak:5 (12.5)<br>Wound infection: 1 (2.5)<br>30-day mortality: 1		NR	NR

© 2021 Mederos MA et al. *JAMA Network Open.*

			mortality: 0 90-day mortality: 2 (5)	(2.5) 90-day mortality: 1 (2.5)			
van der Sluis, 2019[17]	Open McKeown v Robot transthoracic with laparoscopic abdominal and open cervical portions	Readmissions: 4 (7.3) LOS (median):16 ICU stay: 1 (median) Grade >2 complications overall: 44 (80) Grade >2 complications directly related to surgery: 44 (80) Pulmonary complications:32 (58) Chylothorax: 12 (22) MACE: 26 (47) 30-day mortality: 0 (0) 60-day mortality: 1 (2) 90-day mortality: 1 (2) Anastomic leak:11 (20) RLN injury: 6 (11) Reoperations: 18 (32.7) Health-related QOL (6wk):	Readmissions: 6 (11.1) LOS (median):14 ICU stay: 1 (median) Grade >2 complications overall: 34 (63) Grade >2 complications directly related to surgery: 32 (59) Pulmonary complications:17 (32) Chylothorax: 17 (31.5) MACE: 17 (22) 30-day mortality: 1 (2) 60-day mortality: 3 (6) 90-day mortality: 5 (9) Anastomotic leak: 13 (24.1) RLN injury: 5 (9) Reoperations: 13 (24.1) Health-related QOL (6wk):		Median follow-up: 40 months for all both arms Median OS not reached in either arm (no differences between arms).<br><br>Median DFS:28 months	Median follow-up: 40 months for all both arms Median OS not reached in either arm (no differences between arms).<br><br>Median DFS: 26 months	

© 2021 Mederos MA et al. *JAMA Network Open.*

		57.6 (50.6-64.6)<br>Physical functioning (6wk): 58.6 (51.1-66)	68.7 (61.5-75.9)<br>Physical functioning (6wk): 69.3 (61.6-76.9)				
Washington, 2019[18]	Robotic vs laparoscopic transhiatal esophagectomy		LOS: 9.9 (4)<br>ICU stay: 1.7 (2.4)<br>Anastomotic leak: 1 (5.6)<br>Clavien Dindo ≥3: 2 (11.1)<br>Mortality: 0 (0)	LOS: 9.8 (4.7)<br>ICU stay: 2.7 (6.1)<br>Anastomotic leak:1 (5.6)<br>Clavien Dindo >3: 1 (5.6)<br>Morality: 1 (5.6)		Median OS not reached in either arm.	
Yang, 2019[19]	Robot McKeown (abd and thoracic portions) vs thoracolaparoscopic McKeown		Matched LOS (med, range): 11 (6-54)<br>ICU stay: 2 (0-15)<br>Reoperation: 4 (1.5)<br>Total complication:122 (45)<br>Pneumonia: 24 (8.9)<br>Pleural effusion:19 (7)<br>Pneumothorax:7 (2.6)<br>Re-intubation/trach: 12 (4.4)<br>Empyema: 9 (3.3)<br>Arrhythmia: 9 (3.3)<br>Cardiac arrest: 0<br>GI bleeding: 0<br>Anastomotic	Matched LOS (med, range): 11 (4-94)<br>ICU stay: 1 (0-61)<br>Reoperation: 9 (3.3)<br>Total complication: 101 (37.3)<br>Pneumonia: 34 (12.5)<br>Pleural effusion: 31 (11.4)<br>Pneumothorax: 11 (4.1)<br>Re- intubation/trach: 12 (4.4)<br>Empyema: 11 (4.1)<br>Arrhythmia: 8 (3)<br>Cardiac arrest: 2 (0.7)<br>GI bleeding: 1 (0.4)<br>Anastomotic leak:39 (14.4)<br>RLN palsy: 41		Matched<br>N: 255<br>Total recurrence: 30 (11.8)<br>Locoregional recurrence only:9 (3.5)<br>Distal recurrence: 17 (6.7)<br>Locoregionaland distal: 4 (1.6)<br>Mediastinal LN recurrence: 5 (2)<br>Median follow up (med, IQR): 17.2 (1-33)	Matched<br>N: 254<br>Total recurrence: 26 (10.2)<br>Locoregional recurrence only: 10 (3.9)<br>Distal recurrence: 7 (2.8)<br>Locoregionaland distal: 9 (3.6)<br>Mediastinal LN recurrence: 13 (5.3)<br>Median follow up (med, IQR): 9.3 (1-33)

© 2021 Mederos MA et al. *JAMA Network Open.*

			leak: 32 (11.8)<br>RLN palsy: 79 (29.2)<br>Wound infection: 2 (0.7)<br>Chyle leak: 4 (1.5)<br>90-day mortality: 0	(15.1)<br>Wound infection: 2 (0.7)<br>Chyle leak: 2 (0.7)<br>90-day mortality: 2 (0.7)			
Yun, 2019[20]	Open (Ivor Lewis 54.4%; McKeown 45.6%) vs robot-assisted (Ivor Lewis 57.1%; McKeown 42.9%) (abdominal portion was either robot-assisted or laparoscopic)	Unadjusted LOS: 18.2 (15.4)<br>ICU stay: 1.36 (1.97)<br>30 day mortality: 4 (1.7)	Unadjusted LOS: 16.5 (9.8)<br>ICU stay: 1.08 (0.43)<br>30 day mortality: 0 (0)		IPTW-Adjusted 1-year disease-free survival: 53.2%<br>3-year disease-free survival: 45.6%	IPTW-Adjusted 1-year disease-free survival: 54.4%<br>3-year disease-free survival: 49.2%	
Zhang, 2019[21]	Robot-assisted Ivor-Lewis (abdomen and thorax robot) vs thoracolaparoscopic Ivor-Lewis		LOS (med, IQR): 9 (8-12.3)<br>Total complications:19 (28.8)<br>Pneumonia: 4 (6.1)<br>Chylothorax: 0<br>Anastomotic leak: 5 (7.6)<br>RLN palsy: 4 (6.1)<br>MACE: 5 (7.6)<br>Wound infection: 1 (1.5)In-hospital mortality: 0<br>90-day mortality: 1 (1.5)	LOS (med, IQR): 9 (8-11.3)<br>Total complications: 16 (24.2)<br>Pneumonia: 5 (7.6)<br>Chylothorax: 1 (1.5)<br>Anastomotic leak:3 (4.5)<br>RLN palsy: 3 (4.5)<br>MACE: 2 (3)<br>Wound infection: 0<br>In-hospital mortality: 0<br>90-day mortality: 1 (1.5)		NR	NR

eTable 2. Operative Techniques of Included Studies

Author, year	Surgical Approach	Technique	Abdomen	Chest	Anastomosis
Chao, 2018[1]	RAMIE	McKeown	Laparoscopic	Robotic	Circular stapled; cervical
	VAMIE	McKeown	Laparoscopic	VATS	Circular stapled; cervical
Chen, 2019[2]	RAMIE	McKeown	NR	Robotic	Circular stapled; cervical
	VAMIE	McKeown	Laparoscopic	VATS	Circular stapled; cervical
Deng, 2018[3]	RAMIE	McKeown	Robotic	Robotic	Circular stapled or handsewn; cervical
	VAMIE	McKeown	Laparoscopic	VATS	Circular stapled or handsewn; cervical
Espinoza-Mercado, 2019[4]	RAMIE	NR	NR	NR	NR
	VAMIE	NR	NR	NR	NR
	Open	NR	NR	NR	NR
Gong, 2020[5]	RAMIE	McKeown	Robotic	Robotic	Circular stapled; cervical
	VAMIE	McKeown	Laparoscopic	VATS	Circular stapled; cervical
	Open	McKeown	Laparotomy	Thoracotomy	NR
He, 2018[6]	RAMIE	McKeown	Robotic	Robotic	End to side circular stapled; cervical
	VAMIE	McKeown	Laparoscopic	VATS	End to side circular stapled; cervical
He, 2020[7]	RAMIE	McKeown	Robotic	Robotic	NR
	VAMIE	McKeown	Laparoscopic	VATS	NR
Jeong, 2016[8]	RAMIE	McKeown	Laparotomy	Robotic	Cervical
	Open	Ivor Lewis or McKeown	Laparotomy	Thoracotomy	Cervical or thoracic
Meredith, 2019[9]	RAMIE	Ivor Lewis	NR	NR	NR
	VAMIE	Ivor Lewis	NR	NR	NR
	Open	Ivor Lewis	NR	NR	NR
Motoyama, 2019[10]	RAMIE	Ivor Lewis	NR	Robotic	NR
	VAMIE	Ivor Lewis	NR	VATS	NR
Naffouje, 2019[11]	RAMIE	Ivor Lewis	NR	NR	NR
	VAMIE	Ivor Lewis	NR	NR	NR

© 2021 Mederos MA et al. *JAMA Network Open.*

Osaka, 2018[12]	RAMIE	NR	NR	Robotic	NR
	Open	NR	NR	Thoracotomy	NR
Park, 2016[13]	RAMIE	90% McKeown<br>10% Ivor Lewis	58% robotic<br>42% open*	Robotic	90% cervical<br>10% thoracic
	VAMIE	81% McKeown<br>19% Ivor Lewis	49% laparoscopic<br>51% open*	Laparoscopic	81% cervical<br>19% thoracic
Rolff, 2017[14]	Hybrid	Ivor Lewis	Robotic	Thoracotomy	NR
	Open	Ivor Lewis	Laparotomy	Thoracotomy	NR
Sarkaria, 2019[15]	RAMIE	62/64 Ivor Lewis;<br>2/64 McKeown	NR	NR	NR
	Open	103/106 open Ivor Lewis;<br>3/106 thoracoabdominal	NR	NR	NR
Tagkalos, 2019[16]	RAMIE	Ivor Lewis	Robotic	Robotic	Circular stapled; intrathoracic
	VAMIE	Ivor Lewis	Laparoscopic	VATS	Circular stapled; intrathoracic
van der Sluis, 2019[17]	RAMIE	McKeown	Laparoscopic	Robotic	End to side handsewn; cervical
	Open	McKeown	Laparotomy	Thoracotomy	End to side handsewn; cervical
Washington, 2019[18]	RAMIE	Transhiatal	Robotic	NA	Cervical
	VAMIE	Transhiatal	Laparoscopic	NA	Cervical
Yang, 2019[19]	RAMIE	McKeown	Robotic	Robotic	Cervical
	VAMIE	McKeown	Laparoscopic	VATS	Cervical
Yun, 2019[20]	RAMIE	57.1% Ivor Lewis<br>42.9% McKeown	Robotic or Laparoscopic	Robotic	Circular stapled; cervical
	Open	54.4% Ivor Lewis<br>45.6% McKeown	Laparotomy	Thoracotomy	Circular stapled; cervical
Zhang, 2019[21]	RAMIE	Ivor Lewis	Robotic	Robotic	End to end both circular stapled + handsewn; intrathoracic
	VAMIE	Ivor Lewis	Laparoscopic	VATS	End to end circular stapled; intrathoracic

© 2021 Mederos MA et al. *JAMA Network Open.*

eTable 3. Covariates for Propensity Matching in Studies Included in the Meta-analysis

Study	Age	Sex	BMI, height, or weight	Comorbidity/ ASA	Tumor location	Neoadjuvant treatment	Clinical stage	Pathologic stage	Other covariates
Chao et al,[39] 2018	X	X		X		X	X		Recurrent laryngeal nerve nodal findings on preoperative computed tomography imaging
Chen et al,[40] 2019	X	X	X	X		X	X		History of smoking or alcohol consumption
Deng et al,[41] 2019	X	X	X	X		N/A	X		
Espinoza-Mercado,[42] 2019	X	X		X	X	X	X		Facility type, race, insurance type, median income quartile, education level, urban or rural status, year of diagnosis, histology, tumor size
He et al,[45] 2018	X	X	X	X	X	N/A		X	Forced expiratory volume in 1 second
Naffouje,[49] 2019	X	X	X	X		X			Significant weight loss (>10% in 6 months), type of malignancy
Tagkalos,[53] 2019	X		X	X	X	X	X		
Yang et al,[56] 2019	X	X	X		X	X	X	X	
Zhang,[58] 2019	X	X	X	X	X	N/A		X	Tumor size

© 2021 Mederos MA et al. *JAMA Network Open.*

eTable 4. Publication Bias

	**Egger regression test (p-value)**	**Begg rank correlation test (p-value)**
Lymph node harvest	0.712	0.920
EBL	0.442	0.275
Anastomatic leak	0.431	0.260
recurrent laryngeal nerve palsy	0.169	0.381
pulmonary complications	0.590	0.612
total complications	0.330	0.719
mortality	0.892	0.920

© 2021 Mederos MA et al. *JAMA Network Open.*

eTable 5. Risk of Bias

Risk of Bias (randomized trials)

Author, year	Random sequence generation	Allocation concealment	Blinding of participants and personnel	Blinding of outcome assessment	Incomplete outcome data	Selective reporting	Other sources of bias
van der Sluis, 2019[17]	O	O	O	•	Short-term: 0<br>Long-term: 0	O	O
He, 2020[7]	O				Short-term: 0<br>Long-term: 0	O	O
0 = low risk of bias; • = risk of bias; • = unknown							

ROBINS-I (observational studies)

Author, year	Confounding	Selection bias	Bias in measurement classification of interventions	Bias due to deviations from intended interventions	Bias due to missing data	Bias in measurement of outcomes	Bias in selection of the reported result	Other source of bias
Chao, 2018[1]	Low	Moderate	Low	Low	Low	Low	Low	n/a
Chen, 2019[2]	Low	Moderate	Low	Low	Low	Low	Low	n/a
Deng, 2019[3]	Low	Low	Low	Low	Low	Low	Low	n/a
Espinoza-Mercado, 2019[4]	Low	Moderate	Moderate	Low	Low	Low	Low	n/a
Gong, 2020[5]	Serious	Serious	Low	Low	Low	Low	Low	n/a
He, 2018[6]	Low	Moderate	Low	Low	Low	Low	Low	n/a
Jeong, 2016[8]	Low	Moderate	Low	Low	Low	Low	Low	n/a

© 2021 Mederos MA et al. *JAMA Network Open.*

Meredith, 2019[9]	Serious	Serious	Low	Low	Low	Low	Low	n/a
Motoyama, 2019[10]	Low	Serious	Low	Low	Low	Low	Moderate	n/a
Naffouje, 2019[11]	Low	Moderate	Low	Low	Low	Low	Low	n/a
Osaka, 2018[12]	Moderate	Serious	Low	Low	Low	Low	Moderate	n/a
Park, 2016[13]	Low	Serious	Low	Low	Short-term outcomes: Low; Long-term outcomes: Serious	Low	Serious	n/a
Rolff, 2017[14]	Serious	Moderate	Low	Low	Low	Low	Moderate	n/a
Sarkaria, 2019[15]	Low	Moderate	Low	Low	Low	Moderate	Low	n/a
Tagkalos, 2019[16]	Low	Moderate	Low	Low	Low	Low	Low	n/a
Washington, 2019[18]	Serious	Moderate	Low	Low	Low	Low	Serious	n/a
Yang, 2019[19]	Low	Low	Low	Low	Short-term outcomes: Low; Long-term outcomes: Serious	Low	Low	n/a
Yun, 2019[20]	Low	Moderate	Low	Low	Short-term outcomes: Low; Long-term outcomes: Serious	Low	Low	n/a
Zhang, 2019[21]	Moderate	Moderate	Low	Low	Low	Low	Low	n/a

© 2021 Mederos MA et al. *JAMA Network Open.*

eFIGURE 1. Forest Plot of Intraoperative Outcomes

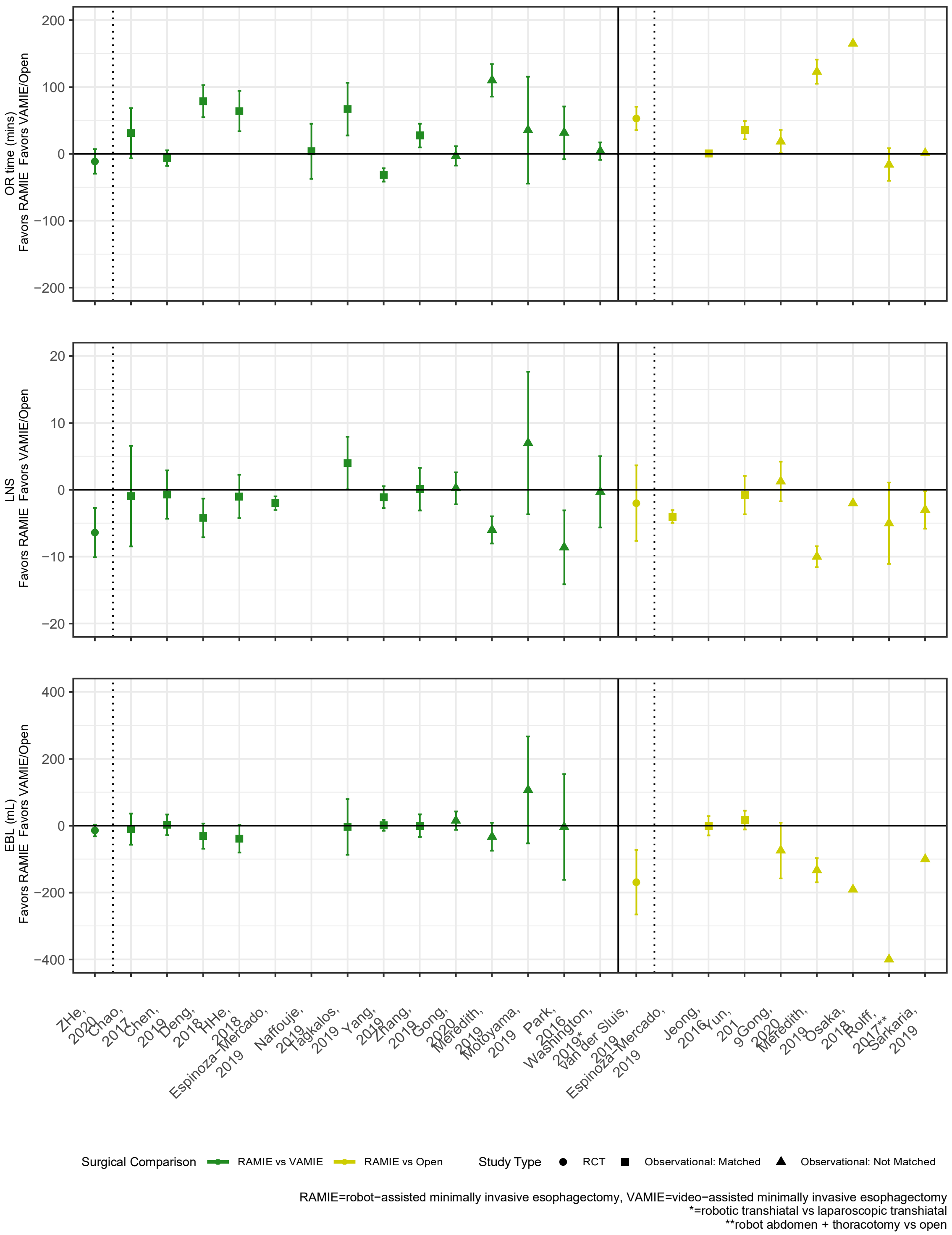

Dashed line: separates randomized trial from observational trials.
Solid line: separates RAMIE vs. VAMIE and RAMIE vs. open esophagectomy comparisons

© 2021 Mederos MA et al. *JAMA Network Open.*

eFIGURE 2. Forest Plot of Short-term Outcomes

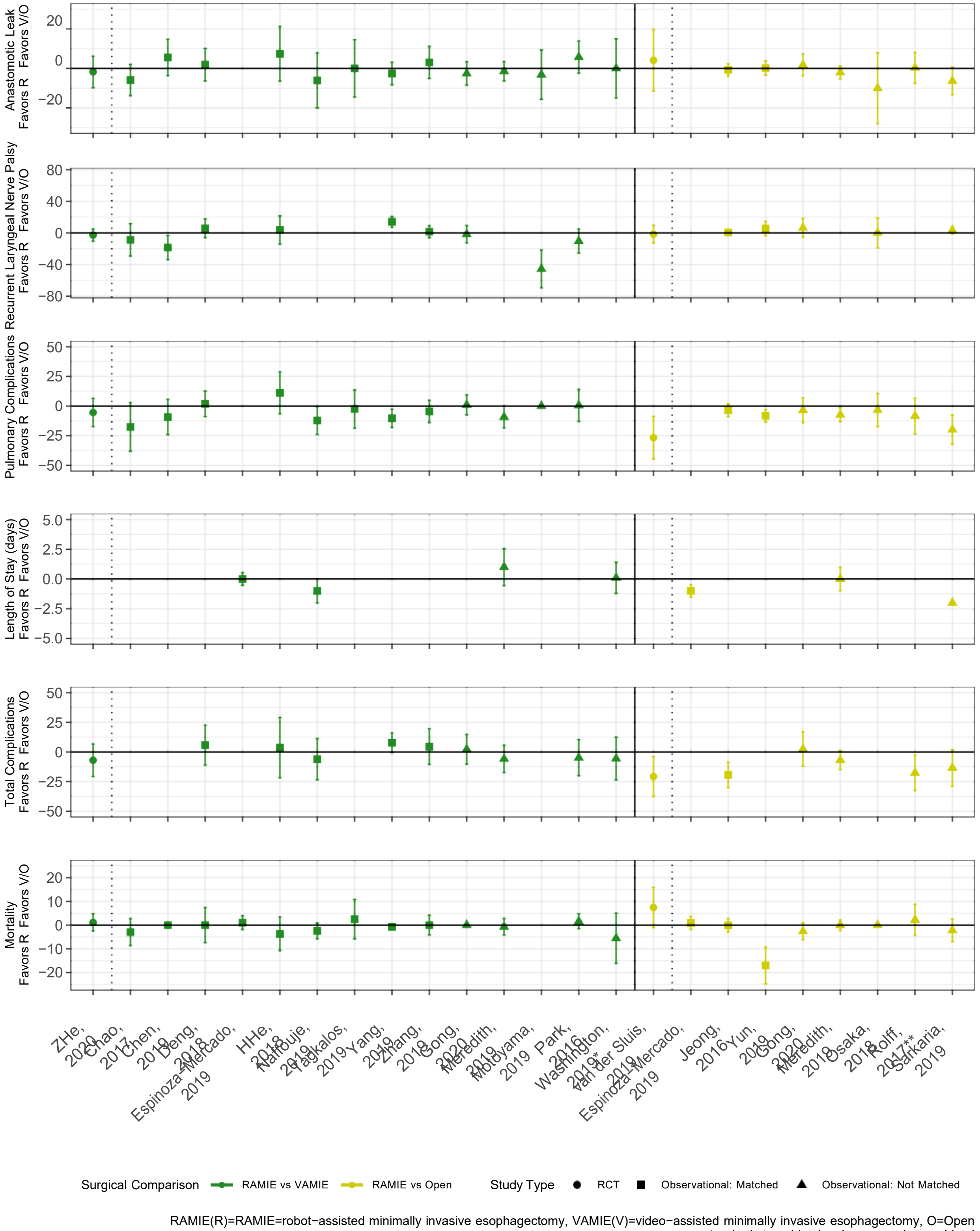

RAMIE(R)=RAMIE=robot−assisted minimally invasive esophagectomy, VAMIE(V)=video−assisted minimally invasive esophagectomy, O=Open
*=robotic transhiatal vs laparoscopic transhiatal
**robot abdomen + thoracotomy vs open

Dashed line: separates randomized trial from observational trials.
Solid line: separates RAMIE vs. VAMIE and RAMIE vs. open esophagectomy comparisons

© 2021 Mederos MA et al. *JAMA Network Open.*

eFIGURE 3. Forest Plot of Long-Term Outcomes

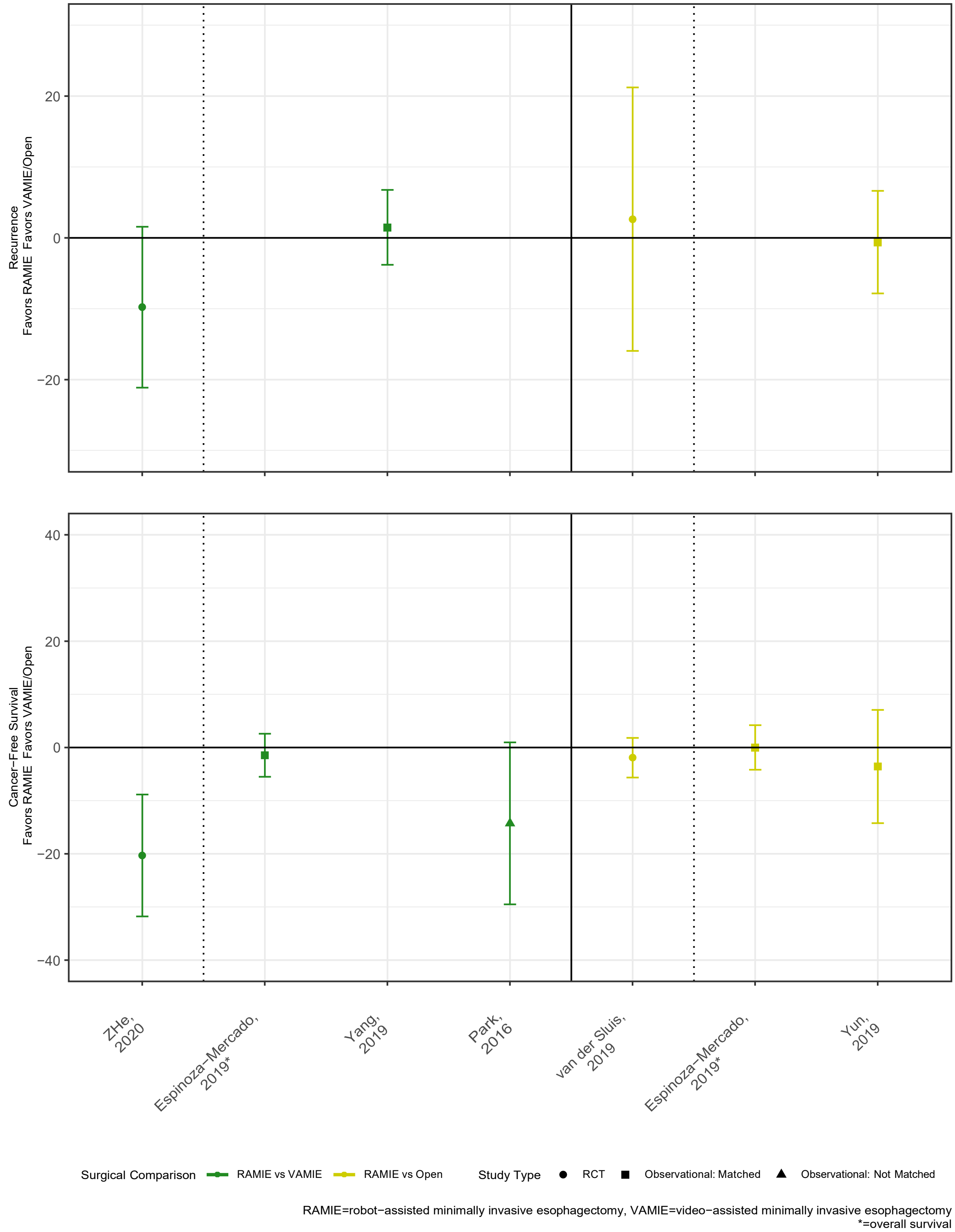

Dashed line: separates randomized trial from observational trials.
Solid line: separates RAMIE vs. VAMIE and RAMIE vs. open esophagectomy comparisons

© 2021 Mederos MA et al. *JAMA Network Open.*

References:

1. Chao YK, Hsieh MJ, Liu YH, Liu HP. Lymph Node Evaluation in Robot-Assisted Versus Video-Assisted Thoracoscopic Esophagectomy for Esophageal Squamous Cell Carcinoma: A Propensity-Matched Analysis. *World J Surg.* 2018;42(2):590-598.
2. Chen J, Liu Q, Zhang X, et al. Comparisons of short-term outcomes between robot-assisted and thoraco-laparoscopic esophagectomy with extended two-field lymph node dissection for resectable thoracic esophageal squamous cell carcinoma. *J Thorac Dis.* 2019;11(9):3874-3880.
3. Deng HY, Luo J, Li SX, et al. Does robot-assisted minimally invasive esophagectomy really have the advantage of lymphadenectomy over video-assisted minimally invasive esophagectomy in treating esophageal squamous cell carcinoma? A propensity score-matched analysis based on short-term outcomes. *Dis Esophagus.* 2019;32(7).
4. Espinoza-Mercado F, Imai TA, Borgella JD, et al. Does the Approach Matter? Comparing Survival in Robotic, Minimally Invasive, and Open Esophagectomies. *Ann Thorac Surg.* 2019;107(2):378-385.
5. Gong L, Jiang H, Yue J, et al. Comparison of the short-term outcomes of robot-assisted minimally invasive, video-assisted minimally invasive, and open esophagectomy. *J Thorac Dis.* 2020;12(3):916-924.
6. He H, Wu Q, Wang Z, et al. Short-term outcomes of robot-assisted minimally invasive esophagectomy for esophageal cancer: a propensity score matched analysis. *J Cardiothorac Surg.* 2018;13(1):52.
7. He ZF, Zheng TL, Liu DL, et al. [Comparison of short-term and long-term efficacy between robot-assisted and thoracoscopy-laparoscopy-assisted radical esophageal cancer surgery]. *Zhonghua Wei Chang Wai Ke Za Zhi.* 2020;23(4):390-395.
8. Jeong DM, Kim JA, Ahn HJ, Yang M, Heo BY, Lee SH. Decreased Incidence of Postoperative Delirium in Robot-assisted Thoracoscopic Esophagectomy Compared With Open Transthoracic Esophagectomy. *Surg Laparosc Endosc Percutan Tech.* 2016;26(6):516-522.
9. Meredith KL, Maramara T, Blinn P, Lee D, Huston J, Shridhar R. Comparative Perioperative Outcomes by Esophagectomy Surgical Technique. *J Gastrointest Surg.* 2019.
10. Motoyama S, Sato Y, Wakita A, et al. Extensive Lymph Node Dissection Around the Left Laryngeal Nerve Achieved With Robot-assisted Thoracoscopic Esophagectomy. *Anticancer Res.* 2019;39(3):1337-1342.
11. Naffouje SA, Salloum RH, Khalaf Z, Salti GI. Outcomes of Open Versus Minimally Invasive Ivor-Lewis Esophagectomy for Cancer: A Propensity-Score Matched Analysis of NSQIP Database. *Ann Surg Oncol.* 2019;26(7):2001-2010.
12. Osaka Y, Tachibana S, Ota Y, et al. Usefulness of robot-assisted thoracoscopic esophagectomy. *Gen Thorac Cardiovasc Surg.* 2018;66(4):225-231.
13. Park S, Hwang Y, Lee HJ, Park IK, Kim YT, Kang CH. Comparison of robot-assisted esophagectomy and thoracoscopic esophagectomy in esophageal squamous cell carcinoma. *J Thorac Dis.* 2016;8(10):2853-2861.
14. HC. R, RB. A, M. B, et al. Robot-assisted hybrid esophagectomy is associated with a shorter length of stay compared to conventional transthoracic esophagectomy, a

retrospective study. http://wwwwhoint/trialsearch/Trial2aspx?TrialID=DRKS00013023. 2017.

15. Sarkaria IS, Rizk NP, Goldman DA, et al. Early Quality of Life Outcomes After Robotic-Assisted Minimally Invasive and Open Esophagectomy. *Ann Thorac Surg.* 2019;108(3):920-928.
16. Tagkalos E, Goense L, Hoppe-Lotichius M, et al. Robot-assisted minimally invasive esophagectomy (RAMIE) compared to conventional minimally invasive esophagectomy (MIE) for esophageal cancer: a propensity-matched analysis. *Dis Esophagus.* 2019.
17. van der Sluis PC, van der Horst S, May AM, et al. Robot-assisted Minimally Invasive Thoracolaparoscopic Esophagectomy Versus Open Transthoracic Esophagectomy for Resectable Esophageal Cancer: A Randomized Controlled Trial. *Ann Surg.* 2019;269(4):621-630.
18. Washington K, Watkins JR, Jay J, Jeyarajah DR. Oncologic Resection in Laparoscopic Versus Robotic Transhiatal Esophagectomy. *Jsls.* 2019;23(2).
19. Yang Y, Zhang X, Li B, et al. Short- and mid-term outcomes of robotic versus thoraco-laparoscopic McKeown esophagectomy for squamous cell esophageal cancer: a propensity score-matched study. *Dis Esophagus.* 2019.
20. Yun JK, Chong BK, Kim HJ, et al. Comparative outcomes of robot-assisted minimally invasive versus open esophagectomy in patients with esophageal squamous cell carcinoma: a propensity score-weighted analysis. *Dis Esophagus.* 2019.
21. Zhang Y, Han Y, Gan Q, et al. Early Outcomes of Robot-Assisted Versus Thoracoscopic-Assisted Ivor Lewis Esophagectomy for Esophageal Cancer: A Propensity Score-Matched Study. *Ann Surg Oncol.* 2019;26(5):1284-1291.

© 2021 Mederos MA et al. *JAMA Network Open.*
